# The emerging role of robotics in plastic and reconstructive surgery: a systematic review and meta-analysis

**DOI:** 10.1007/s11701-024-01987-7

**Published:** 2024-06-15

**Authors:** Laura Awad, Benedict Reed, Edward Bollen, Benjamin J. Langridge, Sara Jasionowska, Peter E. M. Butler, Allan Ponniah

**Affiliations:** 1 Charles Wolfson Centre of Reconstructive Surgery, University College London, Royal Free Hospital, London, UK; 2 Department of Plastic Surgery, Royal Free Hospital, London, UK; 3 Department of Surgery and Interventional Sciences, University College London, Royal Free Hospital, London, UK

**Keywords:** Robotic, Robotic assisted, Plastic and reconstructive surgery

## Abstract

The role of robotics has grown exponentially. There is an active interest amongst practitioners in the transferability of the potential benefits into plastic and reconstructive surgery; however, many plastic surgeons report lack of widespread implementation, training, or clinical exposure. We report the current evidence base, and surgical opportunities, alongside key barriers, and limitations to overcome, to develop the use of robotics within the field. This systematic review of PubMed, Medline, and Embase has been conducted in accordance with the Preferred Reporting Items for Systematic Reviews and Meta-Analyses (PROSPERO (ID: CRD42024524237). Preclinical, educational, and clinical articles were included, within the scope of plastic and reconstructive surgery. 2, 181, articles were screened; 176 articles met the inclusion criteria across lymph node dissection, flap and microsurgery, vaginoplasty, craniofacial reconstruction, abdominal wall reconstruction and transoral robotic surgery (TOR). A number of benefits have been reported including technical advantages such as better visualisation, improved precision and accuracy, and tremor reduction. Patient benefits include lower rate of complications and quicker recovery; however, there is a longer operative duration in some categories. Cost presents a significant barrier to implementation. Robotic surgery presents an exciting opportunity to improve patient outcomes and surgical ease of use, with feasibility for many subspecialities demonstrated in this review. However, further higher quality comparative research with careful case selection, which is adequately powered, as well as the inclusion of cost-analysis, is necessary to fully understand the true benefit for patient care, and justification for resource utilisation.

**Supplementary Information:**

The online version contains supplementary material available at 10.1007/s11701-024-01987-7.

## Introduction

The role of robotics has grown exponentially. Robotic surgery, also known as robotic-assisted surgery, allows for complex minimally invasive surgical procedures to be completely or part-performed with a mechanical system consisting of articulating arms, typically controlled at a separate console by the surgeon.

The Da Vinci Surgical Robotic System (Intuitive Surgical, Sunnyvale, CA, USA), has been widely implemented in various surgical specialities, such as general surgery, urology, and gynaecology, within 66 countries. A recent systematic review of laparoscopic and robotic surgery found comparable or improved complication rates with robotic surgery, with reduced recovery time and length of stay [1].

Robotic consoles can offer accuracy, and precision, as well as minimally invasive access to difficult areas, with improved visualisation. Surgeons have better ergonomic performance, with a reduction in mental and physical workload [2]. Additionally, wireless connection broadens opportunities within telesurgery to facilitate remote operating [3].

**Fig. 1:**
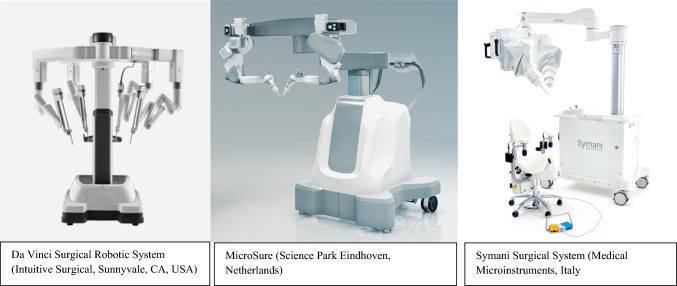
Robotic equipment utilised within clinical practice. All three are controlled by a separate master console

The application of robotic surgery in clinical plastic and reconstructive practice is yet to be well established [4]. There is an active interest amongst practitioners in the transferability of these potential benefits into a speciality that works in collaboration with many surgical disciplines; however, many plastic surgeons report lack widespread implementation or exposure [5]. Whilst Da Vinci Surgical Robotic System (Intuitive Surgical, Sunnyvale, CA, USA) is the most well-known resource, MUSA Microsure (Science Park Eindhoven, Netherlands) and Symani Surgical System (Medical Microinstruments, Italy) are competitors in the market, particularly for use within microsurgery (Fig. 1).

Microsurgery is an area which requires high precision, excellent magnified visualisation, and tremor reduction. Whilst robotic surgery may exceed in these domains, the impact of loss of haptic feedback requires investigation. There are other potential barriers within the widespread implementation of robotics and robotic-assisted surgery within plastic and reconstructive surgery such as the financial incurrence and sparce training opportunities [5].

The aims of this systematic review are to assess the feasibility of robotic surgery within plastic and reconstructive surgery and review the barriers and limitations to clinical implementation and training.

## Methods

This systematic review has been conducted in accordance with the Preferred Reporting Items for Systematic Reviews and Meta-Analyses (PRISMA) [6]. Methodology was designed a priori, and this review is registered with PROSPERO (ID: CRD42024524237).

A literature search of PubMed, Medline and Embase for publications within the past 10 years was conducted by author L.A. Additional articles found through reference screening were included. Titles and abstracts were screened by two independent authors (B.R and E.B), with discrepancies for inclusion reviewed by a third author independently (L.A). This review includes all study types such as randomised controlled trials (RCT), prospective cohort, retrospective cohort, case series/reports, case–control, cross-sectional studies and preclinical studies.

### Eligibility criteria

Articles were accepted for inclusion using the following criteria:

- Patients/populations who have undergone robotic surgery for reconstruction or oncological resection, within the scope of plastic and reconstructive surgery.
- Adults and children
- Articles which described robotic procedures within the scope of plastic and reconstructive surgery
- Preclinical and educational studies within the scope of robotic plastic and reconstructive surgery including animal, synthetic and cadaveric models.

Articles were excluded from this review using the following criteria:

- Articles pertaining to robotic surgery outside the scope of plastic and reconstructive surgery.
- Inguinal hernia repair
- Articles not available in English language
- Articles published prior to 2013.

### Search strategy

Search strategy employed is described below. Key words and subject headings were combined using Boolean logic and refined with consensus from all authors:

- Robot* AND
- Micro* OR reconstruct* OR flap OR nerve OR anastomosis OR abdominal wall OR pelvic floor OR supermicrosurg* OR head and neck OR oral OR oropharyngeal OR vaginoplasty OR breast OR nasal OR plastic

### Data metrics

Data were tabulated into a predetermined Excel spreadsheet by authors LA and E.B [7]. This was subsequently refined following a pilot collection with a random sample of papers. Articles upon paper review which were deemed not suitable for inclusion were discussed with an independent third party (B.L). Data items obtained included article characteristics (title, author, year, journal, impact factor, type of study, multicentre/single centre), demographics (number of participants, gender, age, control), procedure (subspeciality, specific task, robot, ports, location of ports), and outcomes (operative duration, length of Stay, blood loss, peri-operative complications, long-term outcomes, follow-up duration, learning curve, and cost).

### Risk of bias

Risk of bias was assessed by authors LA and E.B. RCT’s were reviewed using Cochrane’s risk of bias tool (RoB 2) [8]. Non-randomised trials was assessed using Cochrane’s ROBINS-I tool [9]. The Joan Briggs Institute Critical Appraisal Checklist for Case Series and the Joan Briggs Institute Critical Appraisal Checklist for Case Reports was used to review case series and case reports, respectively. [10, 11] A report of bias is included in the appendices.

### Data synthesis

Narrative synthesis, and quantitative analysis was performed where possible. Descriptive analysis of continuous data is represented with ranges, mean values, or overall rate. Categorical data is presented with percentage prevalence. Subcategories are defined by subspeciality and procedure.

Study characteristics were tabulated and compared against planned subgroups to determine their suitability for each synthesis. Nonparametric data were analysed using a Wilcoxon test or an unpaired *T* test. Forrest plots were constructed, (in subcategories with article number > 5, where possible), using odds ratios for dichotomous and continuous outcomes and heterogeneity tested for using Chi-square and *I*^2^ test. Statistical analysis was performed using RevMan Software [12].

## Results

**Fig. 2:**
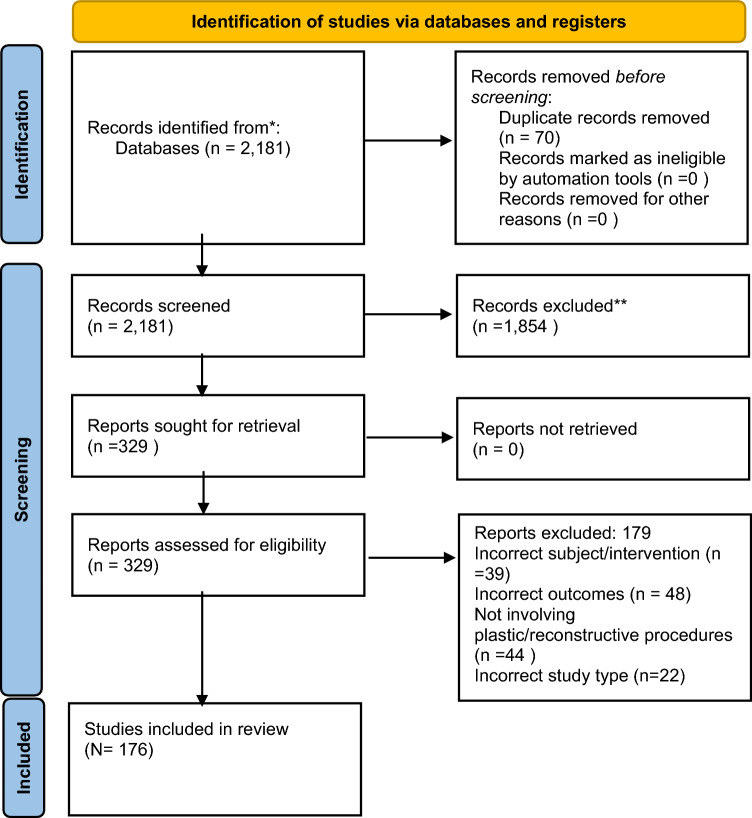
PRISMA flow diagram of the literature search for robotics in plastic and reconstructive surgery

The literature search yielded a total of 2181 articles (Fig. 2). Following abstract screening, a total of 176 articles were included in this systematic review. A total of 149 clinical articles were found (Table 1). A total of 11 preclinical articles were included (Two of which also included clinical data) (Table 2) [13–23]. A total of 18 educational articles were included (Table 2) [24–41].

**Table 1:** Clinical publications within the scope of robotic plastic and reconstructive surgery (*BL* blood loss, *LOS* length of stay)

Reference	Year	N =	Robot	Specific skill/task	Control procedure	Outcomes	Duration of surgery compared to control	LOS compared to control	Peri-operative complications difference compared to control	Length of f/u (months)	Additional outcomes
Lymph Node Dissection = 11 Articles											
Kim et al	2013	20	Da Vinci	Neck dissection	Conventional	Operative time, BL Complications, LOS Number of nodes	Significantly higher	No difference	No difference	8	Higher scar satisfaction with robotic patients
Tae et al	2013	11	Da Vinci	Neck dissection	Conventional	Operative time Post-operative drainage Cosmetic satisfaction	Significantly higher		No difference		Higher cosmetic satisfaction with robotic patients
Kim et al	2015	2	Da Vinci S	Neck dissection	Endoscopic neck dissection	Operative time Complications Scarring Lymphoedema	Higher		No lymphoedema		Excellent cosmetic satisfaction, hidden scar
Du et al	2017	1	Da Vinci	Axillary dissection		Operative time, LOS Complications Number of nodes				10	
Lira et al	2017	6	da Vinci Si	Neck dissection	Endoscopic and conventional	Operative time Complications, LOS Number of nodes Disease-free survival	Higher		Statistically lower	18.6	No difference in resection outcomes or disease-free survival
Melly et al	2017	1	Da Vinci	Axillary dissection		LOS, Complications					
He et al	2018	13	Da Vinci	Axillary dissection		Operative time, BL Number of nodes Complications				16.5	
Singh et al	2018	51	Da Vinci si	Inguinal dissection	Conventional inguinal dissection	Operative time, LOS, BL Number of Nodes Complications	Significantly higher (excluding docking time)	Significantly shorter	Significantly lower incidence of major complications, edge necrosis, limb oedema	40	
Paek et al	2019	28	Da Vinci	Neck dissection	Conventional neck dissection	Complications Number of Nodes	Significantly higher	No difference	No statistical difference		
Lee et al	2020	1	Da Vinci Xi	Axillary dissection		Operative time, LOS Complications Number of Nodes			none		
Song et al	2020	4	Da Vinci	Neck dissection		Operative time Blood Loss Complications					
Craniofacial = 2 Articles											
Lin et al	2022	15	KUKA robotic arm	Mandibular contouring		Osteotomy surface position error/plane angle error Operation time BL, LOS, Complications Safety outcomes Patient satisfaction/pain scale/score at 1&6 months	No statistical difference	No difference	No difference		
Lin et al	2023	15	KUKA robotic arm	Mandibular contouring	Conventional	Osteotomy surface position error/plane angle error Operation time BL, LOS, Complications Safety outcomes Patient satisfaction/pain scale/score at 1&6 months	No difference	No difference	No difference	6	
Cleft Palate = 1 Article											
Teblick et al	2023	29	Da Vinci	Modified Furlow double-opposing Z-palatoplasty		Eustachian Tube Function					Lower hearing thresholds and faster resolution of OME were found
Pedicled and Free Flap Harvest = 21 Articles											
Pederson et al	2014	10	Da Vinci	RAM		Operative time Complications				12	No recurrence
Clemens et al	2014	12	Da Vinci	Pedicled LD	Conventional	Operative time, LOS Complications				7.1	
Chung et al	2015	12	Da Vinci	Pedicled LD		Operative time, LOS Complications Patient satisfaction				15.7	High patient satisfaction with 1–10 Likert scale
Lai et al	2018	1	Da Vinci	Pedicled LD		Complications				5	
Lai et al	2018	2	Da Vinci Si	Pedicled LD		Operative time Complications Blood Loss Technical report				8	
Houvenaeghal et al	2019	80	Da Vinci Si /Xi	Pedicled LD		Operative time, LOS Complications, BL					Lack of long dorsal scar
Ozkan et al	2019	1	da Vinci Xi	Omental		Operative time Complications Flap Survival				12	
Houvenaegel et al	2020	46	da Vinci Xi/Si	Pedicled LD	Conventional	Operative time, LOS Complications	Significantly higher (improved with experience)	No difference	Significantly lower		
Fouarge et al	2020	6	da Vinci si /Xi	Pedicled LD		Operative time, LOS Complications Technical report					
Frey et al	2020	5	da Vinci xi	Free omental flap (lymph node transfer)		LOS, Complications					
Haverland et al	2020	6	Da Vinci	RAM (pelvic recon)		Complications				9.2	
Moon et al	2020	21	da Vinci xi	Pedicled LD		Operative time Complications				19.2	
Winocour et al	2020	25	Da Vinci	Pedicled LD	Conventional	Operative time, LOS Complications Flap Survival	Significantly higher	Significantly shorter	Higher rate of seroma	60	Reduction of opioid requirements but not significant
Asaad et al	2021	7	Da Vinci	RAM (pelvic reconstruction)		LOS, BL Complications					
Day et al	2021	1	Da Vinci	Pedicled omental		Complications					
Joo et al	2021	1	Da Vinci SP	Pedicled LD		Histology Operative time Complications PROMS—BREAST Q					
Cheon et al	2022	41	da Vinci Si/Xi/SP	Pedicled LD		Operative time, LOS Complications				15.6	Higher patient satisfaction with robotic group
Davila et al	2022	16	Da Vinci	RAM (pelvic reconstruction)	Conventional	Operative time, LOS Complications	No difference	No difference	No difference statistically but lower rate in robotic group	36	
Hwang et al	2022	3	da Vinci SP	Pedicled LD		Operative time Complications					
Seon Eo et al	2023	20	Da Vinci	Pedicled LD	Endoscopic conventional	Operative time, LOS Opioid requirement Complications	Significantly higher	No difference	No difference	18.4	statistically higher overall patient satisfaction
Shin et al	2023	11	da Vinci si	RFFF harvest		Operative time Method of anastomosis Flap Survival Complications	Significantly longer				Notably less scarring with no longitudinal volar incision
Hans et al	2013	2 (1 flap inset)	Da Vinci	RFF inset, TOR resection		Operative time, LOS Resection margins				8	
Flap Inset and Anastomosis (Vessel, Nerve. Lymph) = 16 Articles											
Song et al	2013	5	Da Vinci	Flap inset, anastomosis, TOR resection		Operative time Complications				12.8	
Lai et al	2014	5	da Vinci Si	RFF inset, venous anastomosis		Operative time Complications Flap Loss				7	
Miyamato et al	2014	6	da Vinci S	Nerve graft		Complications Deltoid function recovery				10	5/6 regained deltoid function
Tsai et al	2017	14	Da Vinci	RFF inset, secondary venous anastomosis	Conventional	Complications Flap Revision Long Term Outcome (FIGS)			No difference	3	
Lai et al	2019	15	Da Vinci	RFF anastomosis (vein/artery)	Microscope (conventional)	Operative duration Complications Vessel Diameter	Significantly higher		100% flap survival	11.5	
Van Mulken et al	2020	20	MUSA	LVA	Microscope (conventional)	Operative time Anastomosis patency Complications Patient satisfaction Postoperative: daily wearing of compressive garment, manual lymph drainage, lymph ICF score, EUL index score	Significantly higher anastomosis time but showed steep improvement with learning curve		No difference	3	No difference in Lymph ICF score
Chang et al	2021	1	da Vinci Xi	Bilateral sympathetic trunk recon with sural nerve graft (neurosynthesis)		LOS PROM (questionnaire)				42	70% improvement in symptoms
Barbon et al	2022	22	Symani surgical system	Arterial/venous/nerve/lymph	Microscope (conventional)	Operative duration Learning curve Suture size Number of sutures Patency	significantly higher BUT improved in second cohort more than hand-sewn				Steep robotic learning curve to a comparable time with hand sewn anastomosis
Lindenblatt et al	2022	5	Symani surgical system	LVA/arterial		Operative Time Surgical Experience					
Van mulken et al	2022	20	MUSA	LVA	Microscope (conventional)	1-year outcomes—QOL (Arm circumference, compression garment, manual drainage, arm dermal backflow and patency of anastomosis tested with ICG				12	Patient outcomes were comparable with conventional procedure. 42.9% reduction in use of compression garments
Beier et al	2023	23	Symani surgical System	Arterial End-to-end/End-to -side anastomosis		Operative Time Revisions					
Besmens et al	2023	6	Symani Surgical System (and exoscope)	Arterial anastomosis/neurosynthesis		Operative time Anastomosis patency					
Chen et al	2023	23	da Vinci xi	Nerve anastomosis		Operative time, LOS Level of nerve injury Length of defect Complications				24	Effective in reducing sweating across all sites assessed
Innocenti et al	2023	1	Symani surgical system	Arterial/venous anastomosis ALT		Surgical Experience Number of sutures Operative Time Complications					
Weinzierl et al	2023	8	Symani surgical system	LVA		Operative Time Number of sutures Anastomotic Patency					
Flap Pedicle Dissection = 8 Articles											
Gundlapalli et al	2018	1	Da Vinci	DIEP		Operative Time Complications Cost				9	
Choi et al	2021	17	da Vinci sp	DIEP		Operative time Complication					
Bishop et al	2022	21	Da Vinci	DIEP		Operative time, LOS Complications				5	
Daar et al	2022	4	da Vinci xi	DIEP		Operative time, LOS Complications				6.31	
Dayaratna et al	2022	1	da Vinci Xi	DIEP submuscular pedicle dissection		Operative time Complications PROM—BREAST Q			None, minimal pain, low analgesic requirements	3	
Wittesaele et al	2022	10	Da Vinci	DIEP		Operative time, LOS Complications				1	
Tsai et al	2023	13	Da Vinci	DIEP	Conventional pedicle dissection	Operative time Complications				14	
Zanaty et al	2023	1	Da Vinci	Internal thoracic artery harvest		Surgical Experience Complications					
Vaginoplasty = 3 Articles											
Boztosun et al	2016	1	Da Vinci Xi	Sigmoid vaginoplasty		Operative time, LOS Complications Long term outcomes				6	More minimally invasive approach
Dy et al	2021	47	2 groups—Da Vinci SP/Xi	Vaginoplasty—gender affirmation	SP vs Xi da Vinci	Operative time Complications, BL Neovaginal dimensions	SP shorter duration	No statistical difference	Not statistically different lower rate of BO in xi, higher rate of transfusion and vaginal stenosis in SP	12	Few incisions result in better cosmetic benefit single port shorter and doesn’t impede second surgeon
Blasdel et al	2023	43	Da Vinci SP	Vaginoplasty with peritoneal flap recon, in patients with genital hypoplasia	Traditional vaginal canal dissection, peritoneal flap	Complications PROMs				12	
Nerve Decompression = 1 Article											
Bruyere et al	2016	1	Da Vinci Si	Neurolysis of lateral cutaneous nerve of the thigh		LOS, Complications	-			6	100% reduction in pain (0/10 from 5/10)
Abdominal Wall Reconstruction = 28 Articles											
Chen et al	2016	39	Da Vinci	Small ventral hernia mesh repair	Laparoscopic	Operative time, LOS Complications Defect size	Significantly longer (65)	No difference	No difference in readmissions, no difference in complication rate	1.5	
Bittner et al	2017	26	da Vinci si/xi	Transversus Abdominis release and mesh	Open	Hermia Characteristics Operative time, LOS Complications	Significantly higher (287)	Significantly shorter (6)	No difference	2	
Gonzalez et al	2017	368	Da Vinci Xi	Ventral wall hernia repair mesh/direct closure		Operative time, LOS Complications					
Jamshidian et al	2017	3	da Vinci Xi	Spigelian hernia repair with mesh		Operative time, BL, LOS Complications Opioid Use					
Prabhu et al	2017	177		Intraperitoneal mesh repair	Laparoscopic	Operative time, LOS Complications	Significantly higher	Significantly shorter	Statistically lower		Higher rate of bowel injury, and systemic problems in laparoscopic group
Wang et al	2017	1	Da Vinci	Stratafix/mesh intercostal hernia repair		LOS, Complications					
Warren et al	2017	53	Da Vinci	Retromuscular/ PP mesh ventral hernia repair	Laparoscopic	Rate of fascial closure Operative time, LOS Opioid use Complications Cost	Significantly longer	Significantly shorter	Significantly higher rate of seroma	2	LVHR and RRVHR ($13,943 vs. $19,532; p = 0.07) Robotic cost of procedure higher, however overall cost comparable as shorter length of hospital stay fascial closure achieved more in robotic surgery, extraperitoneal mesh was performed majority of robotic cases
Carbonell et al	2018	111	Da Vinci	Retromuscular mesh hernia repair	Open	Complications, LOS Readmission/Reoperations	Significantly longer	Significantly shorter	Higher rate of seroma/sso	1	Higher surgical site occurrences were noted with r-RVHR, consisting mostly of seromas not requiring intervention
Martin-Del-Campo et al	2018	38	not specified	Transversus Abdominis release & retromuscular synthetic mesh	Open	Hernia dimensions Operative time, LOS, BL, Complications	Significantly higher (211)	Significantly shorter (6)	Systemic complications significantly lower (0 vs 13)		
Muysoms et al	2018	41	da Vinci xi	Retromuscular TAR mesh umbilical hernia repair		Learning curve Operative time Complications PROM—EuraHS-QoL				1	Total skin-to-skin operative time decreased through the series Significant improvement in PROMS compared to preop scores
Walker et al	2018	142	Da Vinci	Preperitoneal mesh ventral hernia repair	Laparoscopic	Operative time, LOS Complications	Significantly longer	No difference	Significantly lower	4	
Kudsi et al	2019	1	da Vinci Xi	Sugarbaker parastomal hernia repair with mesh and TAR		Operative time, LOS, BL, Complications				3	
Kudsi et al	2020	164	da Vinci xi	Transabdominal vs totally extraperitoneal hernia repair all robotic	Transabdominal vs totally extraperitoneal hernia repair all robotic (all retromuscular)	Operative time, BL, LOS Complications Pain Readmission	TEP shorter duration of surgery	No difference	Minor complications statistically higher for TA group, seroma frequency and rate of SSE also higher	Minor complications statistically higher for TA group, seroma frequency and rate of SSE also higher	
Olavarria et al	2020	65	da Vinci xi	Intraperitoneal mesh ventral hernia repair	Laparoscopic	Operative time, LOS Complications Long term outcomes—Recurrence, QOL, Cost	Significantly longer	No difference	No differences	6.4	Clinicians had 50 cases as a learning curve prior trial Increased cost for robotics of 90 days of care including surgery ($15 865 (£12 746; €14 125) v $12 955; cost ratio 1.21, 1.07 to 1.38; adjusted absolute cost difference $2767, $910 to $4626; P = 0.004)
Mudyanadzo et al	2020	16	not specified	Ventral incisional hernia repair	Laparoscopic	LOS, Opioid use		No difference	Use of opioids reduced for robotic group	2	
Petro et al	2020	39	da Vinci si/xi	Intraperitoneal ventral hernia mesh repair	Laparoscopic	Operative time, LOS Long term outcomes—recurrence, NRS 11 score, PROM ISPI, hernia specific QOL Cost	significantly longer (94)	No difference	No difference	3	Cost of reusables was comparable but higher cost for robotics because of higher operative time Comparable NRS 11 score/PROMS
Bergholz et al	2021	1		Mesh intercostal hernia repair		LOS, Complications					
Dhanani et al	2021	65	da Vinci xi	Intraperitoneal mesh and direct closure repair	Laparoscopic	Complications Long term outcomes—PROM, functional status, VAS, cosmetic satisfaction			Not reported	12	No difference in PROM outcomes at 1 year
Rayman et al	2021	3	da Vinci Si	Transabdominal preperitoneal spigelian hernia repair	Laparoscopic	Operative time, LOS Complications Post op pain Defect/mesh size				17	
costa et al	2022	18	Not specified	Intraperitoneal ventral hernia mesh repair	Laparoscopic	Operative time, LOS Complications Long term outcomes—recurrence, EORTC QLQ-C30	Significantly longer	No difference	No difference	24	No difference in long-term outcomes
Kakela et al	2022	19	da Vinci xi	Retromuscular TEP mesh	laparoscopic	Operative time, LOS Long term outcomes.- pain, VAS (M1, 12), SF-36 PROM, hernia recurrence	Significantly longer	No difference		12	Robotic less pain at 1 month and 1 year using VAS all 9 scores for SF-36 favour robotics but not statistically significant for most emotional status and social function improved significantly
Kudsi et al	2022	138	Da Vinci	Mixed methods ventral hernia repair	Obesity class II vs class III	LOS, Complications		No difference	No difference	33.6	Comparison of patient BMI and outcomes in robotics no difference in peri-operative outcomes and intra-operative variables, except higher rate of mesh use in more obese patients
Petro et al	2022	38	da Vinci si/xi	Intraperitoneal ventral hernia mesh repair	Laparoscopic	LOS, Long term outcomes—pain intensity, PROMIS pain score, HERQless, recurrence, reoperations				12	Higher rate of recurrence but better PROM outcomes
Pereira et al	2022	665		Lateral abdominal mesh hernia repair	Open	Operative time, LOS Complications Long-term outcomes—PROM (HerQless, PROMIS pain,)	Significantly longer	Significantly shorter	Bowel injury higher in open but not significant SSI/use of epidural significantly lower overall significantly lower rate of complications	12	No difference in long-term outcomes
Shimada	2022	1	da Vinci xi	Retromuscular extraperitoneal mesh ventral hernia repair		Operative time, LOS, BL, Complications				7	
Dhanani et al	2023	65	da Vinci xi	Intraperitoneal mesh and direct closure repair	Laparoscopic	Complications Long term outcomes—functional status, VAS, cosmetic satisfaction			No difference	24	Significantly lower rate of revision surgery in robotic group no difference in SSO at 2 years (seroma/haematoma) no difference in PROMS
Lima et al	2023	1		Spigelian hernia mesh repair		Complications				0.25	
Petro et al	2023	100	Da Vinci	Robotic enhanced view totally extraperitoneal (eTEP) or robotic intraperitoneal onlay mesh midline ventral hernia < 7 cm	TEP vs intraperitoneal mesh	LOS, Complications Opioid Use Long term—PROM	Significantly longer for TEMP		No difference between robotic groups	12	Significant difference in HERQLes favouring IPOM (but only 12 months post op) no difference in recurrence at 1 year (51 totally extraperitoneal, 49 intraperitoneal mesh)
Mastectomy = 18 Articles											
Sarfati et al	2017	1	Da Vinci	Nipple-sparing mastectomy		Operative time, LOS				3	
Toesca et al	2017	3	Da Vinci S	Nipple-sparing mastectomy		Operative time, LOS Complications Patient satisfaction				8	No complications at 8 months, high cosmetic satisfaction small incision Reduction in operative time from 7–2.5 h over 3 cases
Toesca et al	2017	29	Da Vinci Xi/Si	Nipple-sparing mastectomy		Learning Curve Operative time Complications, LOS				8	
Lai et al	2018	15	Da Vinci Si	Nipple-sparing mastectomy		Learning Curve Operative time Complications, LOS				6.3	100% high satisfaction reported, shorter duration of surgery with more surgical experience
Park et al	2018	1	Da Vinci Xi	Nipple-sparing mastectomy		Operative time Pathology, LOS Complications			0	12	
Rajappa et al	2018	1	Da Vinci Si	Nipple-sparing mastectomy		Operative time, LOS Complications				Not specified	
Sarfati et al	2018	1	Da Vinci Xi	Nipple-sparing mastectomy		Complications					
Sarfati et al	2018	33	da Vinci Xi	Nipple-sparing mastectomy (immediate implant recon)		Operative time Complications PROMS—BREAST Q				12	
Lai et al	2018	2	da Vinci Si	Nipple-sparing mastectomy & lat dorsi flap harvest		Operative time, LOS Complications			0	8	
Houvenaeghal et al	2019	27 (17 lat dorsi flap)	da vinci Si /Xi	Nipple-sparing mastectomy		Operative time Learning curve Complications, LOS					Time of surgery and anaesthesia decreased with learning curve
Houvenaeghal et al	2019	80	Da Vinci Si /Xi	Latissimus dorsi flap robotic ± mastectomy		Operative time, LOS Complications					Single incision, lack of long of long dorsal scar
Kuo et al	2019	3	da vinci xi	Nipple-sparing/skin-sparing mastectomy		Operative time, LOS Complications				5	
Lai et al	2019	22	Da Vinci	Nipple-sparing mastectomy		Operative time docking time BL, complications recurrence	Docking time dropped with more experience			6.9 ± 3.5	All patients reported to be satisfied with outcome US $6000/ use
Lai et al	2019	39	Da Vinci	Nipple-sparing mastectomy		Operative time Learning curve complications pathology/resection margins				8.6	Significantly reduced surgical duration with procedures performed over 1 year
Lai et al	2020	54	Da Vinci Si	Nipple-sparing mastectomy	Conventional	Operative time, BL Resection margins Complications Cost Long term outcomes -photography, PROMS (cosmesis)	Significantly longer	Significantly longer	no difference	14	$10,877 robotic vs $5702 conventional—significantly higher Significantly higher patient satisfaction (better scar, better nipple position)
Toesca et al	2021	40	Da Vinci S	Nipple-sparing mastectomy	Conventional	Operative time, LOS Complications, BL Long term recurrence, survival, PROMs (breast-Q, NAC questionnaire)	Significantly higher	Significantly shorter	significantly lower (notably nipple ischaemia, skin necrosis, haematoma, seroma, and open group more likely to have > 1 complication	28.6	Significantly higher satisfaction in robotic, and in psychological wellbeing No difference in rate of implant loss
Park et al	2022	167	Da Vinci Si	Nipple-sparing mastectomy	Conventional	Complications Recurrence			significantly lower rate of complications in 30 days including nipple necrosis lower rate of Clive dindo classification 3	18	No difference in recurrence
Moon et al	2022	40	da Vinci S	Nipple-sparing mastectomy	Conventional	Complications Pain	Significantly longer		No difference	Perioperative only	Lower pain reported for robotic group
Transoral Robotic Surgery = 43 Articles											
Chan et al	2013	4	Da Vinci	Resection parapharyngeal space neoplasm		Histopathology Complications, LOS Long term outcomes—function/oral diet			None	1–15 months	
Chia et al	2013	2015		Oropharyngeal carcinoma (majority t1/t2 staging)		Complications					Low rate of long-term PEG dependency
Durmus et al^a^	2013	22	da Vinci S/Si	Resection of cancer of unknown primary oral		Operative time Complications					All achieved oral diet D1 RT 100% of patients 0 trachy, 0 gastrostomy
Durmus et al^b^	2013	3		Retromolar trigone tumour resection HPV neg		Operative time, BL Complications				1–16 months	
Hans et al	2013	2	Da Vinci	T3 hypopharyngeal SCC resection and RFF inset		Operative time, LOS Complications				8	(1 flap inset)
Lee et al	2013	27	Da Vinci	Lateral oropharyngectomy (T1/T3 tonsillar cancer)	Conventional	Operative time, LOS BL, Complications Long term outcomes—survival, VHI, MDADI	Significantly shorter than mandibulotomy significantly longer than transoral	Significantly shorter than mandibulotomy, no difference from transoral	No difference	20.3	Higher disease-free and overall survival compared with control No difference in VHI/MDADI scores
Patel et al	2013	47		Oropharyngeal tumour identification		Resection margins Complications					
Tsang et al	2013	1	Da Vinci S	Nasopharyngectomy via lateral palatal flap approach		Resection margins Operative duration Complications				6	
White et al	2013	64	Da Vinci	Recurrent oropharyngeal SCC resection T1-4	Open	Operative time, LOS Complications, BL Long term—OS, DFS, death	Significantly shorter	Significantly shorter	Significantly fewer (fistula and oedema and overall)	24	Lower rate of tracheostomy/NG tube with robotic surgery Decreased incidence of positive margins with robotic surgery (significant) 2-year recurrence free significantly higher for robotic
Chung et al	2014	641		Partial pharyngectomy	Conventional	LOS, Complications		Significantly shorter	Significantly lower		Lower cost for robotic surgery overall. $29,365 vs $20,706 lower rate of tracheostomy and peg
Chung et al (same as above × 3 diff data sets)	2014	147		Partial glossectomy base of tongue	Conventional	LOS, Complications		Significantly shorter	No difference		Lower overall cost for robotics $19,091 vs $23,414 open Significantly lower rate of tracheostomy and PEG
Chung et al (same as above × 3 diff data sets)	2014	68		Partial glossectomy (anterior)	Conventional	LOS, Complications		Significantly longer	No difference (except higher rates of transient dysphagia)		No difference in total cost ( robotic $22,111 vs open $21,376) Signifiantly lower rate of tracheostomy and PEG
Durmus et al	2014	22		Oral cancer of unknown primary resection		Operative time Complications Long term outcomes—HCNI PROM				12	Patients maintain long-term and highly functional QOL status
Ford et al	2014	65	Da Vinci	OPSCC resection (majority t1/t2)	Conventional	Operative time Resection Margins long-term—OS, DFS				36	Significantly higher 3-year survival for robotic group recurrence free survival
Hammoudi et al	2014	26	Da Vinci	Primary scc resection (any neck dissections were conventional)	Conventional	Operative time Resection margins Length of Stay Complications Tracheostomy prevalence Cost	No difference	Significantly shorter	No difference	19	Significantly fewer tracheostomies (n = 4) SIGNIFICANTLY shorter duration of NG feed No difference in 3-year disease-free survival Significantly lower cost for robotics (higher operative cost ($7781 vs $4375) lower overall cost due to length of stay ($20,885 vs $27,926)
Van Loon et al	2014	18	Da Vinci	T1/T2 OP cancer resection		Operative time Resection Margins Blood loss Long term outcomes -PROMS (EORTC-C30, H&N35)				33.7	2-year disease-free survival of 86%
Almeida et al	2015	410		Larynx/pharyngeal cancer		Resection margins Long term outcomes -OS, DFS				20	
Dabas et al	2015	60	Da Vinci	Resection oropharyngeal ca and conventional neck dissection (ipsilateral)		Operative time Blood Loss Complications Long term—functional outcomes				8	
Mercante et al	2015	13		T1/T2 OP ca without adjuvant Tx		procedure time, set up time, operative time complications hospital stay blood loss recovery to normal breathing swallowing/removal of NGT				12	Long term—QoL, dysphagia score, FESS, penetration aspiration scale, MDADI, VHI-10 @ 6&12 months
Mockelmann et al	2015	41		Oropharyngeal resection T1-4	Staged vs concomitant neck dissection (21 control with concurrent, and 20 in intervention arm)	Length of Stay Complications Timing of neck dissection					Timing of neck dissection didn’t make a difference in outcomes. (immediate vs average of 10 days)
Razafindranaly et al	2015	84	Da Vinci	Supraglottic scc resection		Resection margins Complications Oral diet/tracheostomy prevalence			20—tracheostomy temp 64—NG for median of 8 days (0–10) 8—permanent PEG	14	
Smith et al	2015	42	Da Vinci	oropharyngeal SCC resection and neck dissection (majority t3/4)	CRT ( non-operative)	Resection margins Complications Long term—OS, DFS				36	
Aubry et al	2016	178	Da Vinci	Tumour resection		Length of stay Complications					
Fujiwara et al	2016	10	Da Vinci	OPSCC T1-T2		Operative time Length of Stay Complications Resection margins Surgical feasibility Blood Loss Function (swallow)					
Granell et al	2016	1	Da Vinci S HD	Access and resection of parapharyngeal tumour (cavernous haemangioma)		Resection margins length of Stay Complications			None described	12	
Duek et al	2017	1	Da Vinci	Resection parapharyngeal space tumour		Operative time Complications			None	4	
Frenkel et al	2017	425		H&N resection (333 concurrent neck dissection, 92 staged neck dissection)		Complications Length of Stay		Risk adjusted LOS was less for concurrent			neck dissection timing not associated with changes in complications, readmissions, tracheostomy, or gastrostomy
Gorphe et al	2017	27	Da Vinci Xi	TOR resection		Operative time resection margins complications unplanned tracheostomy/death			15 temp tracheostomies		
Lallemant et al	2017	23	Da Vinci	SCC posterior pharyngeal wall resection		Resection margins Length of Stay Complications Long term—OS, DFS			NG feed needed for average of 22 days × 4 PEG (note post -op dysphagia—most likely due to site)	27	
Mahmoud et al	2017	559		TOR OPSCC vs primary CRT	Primary CRT	Long term outcomes—DFS, OS				29	
Rubek et al	2017	30	Da Vinci Si HD	Oropharyngeal SCC		Length of Stay Resection margins Complications			Tube dependency 4.6 days	19	
Sethia et al	2017	111		OP cancer resection—TOR vs TOR and adjuvant therapy		Complications Long term—PROM (HCNI), OS, recurrence			Reduced rate of PEG at immediately post op 3,6,12 months	12	There were no statistically significant differences (P > .05) in aesthetics, social disruption (attitudinal), or speech (attitudinal) at any time point. Also, there were no statistically significant differences (P > .05) for all QOL domains at 12 months
Alessandrini et al	2018	8—Xi 8—S	da vinci Xi vs S	BOT SCC resection (T1/T2) Da vinci si vs xi	× 2 robotic groups	operative time Resection margins complications blood loss post-operative functional outcomes (VAS, LOS, NG)	Si had statistically longer console time and overall operating time	Significantly shorter for Xi	Xi significantly shorter NG use and pain scores no complications reported	Not specified	
Doazan et al	2018	122		Supraglottic SCC		Long term—OS, DFS, recurrence				42.8	
Li et al	2018	2224		T1/T2 OPSCC resection	Conventional and TLM	Resection margins OS		Significantly shorter		60	TOR not associated with increased survival however there is a lower likelihood of need for CRT "
Scott-Wittenborn et al	2018	6	Da Vinci	Base of tongue/palate unknown primary		ICG intraoperative tumour identification intraoperatively using ICG—unsuccessful					ICG was not beneficial for tumour identification or resection using the da vinci
Hardy et al	2019	1		T3 pharyngeal SCC		resection margins complications recurrence				24	
Nichols et al	2019	34		OPSCC T1-T2	Radiotherapy	Complications Long term outcomes -PROMS, recurrence				25	Higher rate of neutropenia, hearing loss, and tinnitus in radiotherapy group, with a higher rate of trismus reported within the TOR group
Petruzzi et al	2019	1	Da Vinci Si	Retropharyngeal lymph node dissection		Operative time					
Holcomb et al	2020	2	Da Vinci Si	Salvage oropharyngectomy and submental artery island flap inset		Operative time, LOS Complications				4	
Kubik et al	2020	23	Da Vinci	HPV unknown primary—BOT mucosectomy		Surgical experience Complications Long term—survival, recurrence				23	
Sano et al	2021	68		Resection	Conventional and TLM	Resection margins					
D'Andrea et al	2022	53	Da vinci Xi	Salvage surgery oropharyngeal (Mostly T2)		Complications Long term outcomes—PROM (MDADI, EORTC QLQC30/ H&N35				24	The preoperative, 1-year, and 2-year MDADI total scores were 71.4, 64.3, and 57.5, respectively. The preoperative, 1-year, and 2-year QLQ-C30 global scores were 61.2, 59.4, and 80.6, respectively. Decannulation was possible in 97.1% of the tracheotomized patients. The two-year enteral tube dependence was 23.1%. The two-year overall survival, disease-free survival, and local control rates were 59%, 46.1%, and 80.9%, respectively
Nichols et al	2022	34		TOR OPSCC T1-T2	Radiotherapy	Long term outcomes recurrence MDADI QLQ-C30 H&N35 VHI-10 FOIS				45	MDADI 2 years—84.8, 3 years 83.3. no difference in functional outcome
Virgilio et al	2023	139		OPSCC resection and neck dissection (mostly t1/t2)		Resection margins tracheostomy/PEG prevalence Long term—recurrence, OS, DFS				26	TOR can de-intensify the need for CRT

**Table 2:** Preclinical and educational articles within the scope of robotic plastic and reconstructive surgery

Topic	First author	Year	Robot	Model/number	Operation performed	Participant	Outcomes reported
Preclinical – 11 articles							
Lymph node dissection	Lee et al	2020	Da Vinci Xi	Cadaveric *N* = 2	Axillary dissection	Expert surgeon	Safety and feasibility prior to clinical implementation
Microsurgery	Feng et al	2017	Robotic ENT Microsurgical System	Chicken *N* = 7 (each arm) conventional vs Robotic	End-to-end anastomosis	6 novices 1 Expert surgeon	Microvascular tremor scale (based on instrument tip movement) was significantly lower for robotic Comparable duration between conventional and robotic groups Subjective feedback found robotic performance to be more accurate with improved handling and stability
Microsurgery	Van Mulken	2018	Microsure	Silicone vessel/Rat *N* = 8 (Each arm) conventional vs robotic	Preparation, transection, and anastomosis	Expert surgeon	Longer time to complete procedure for robotic (27 vs 12 min) 3 events of system reset required
Microsurgery	Ballestin et al	2020	Symani Surgical System	Synthetic 1 mm vessel (6 manual & 6 robotic performed by each trainee)	Microneedle driving, stitch placement, anastomosis	40 expert surgeon 20 novices	Improved precision with robot in both groups (suture distances, angulation) Longer time to perform anastomosis (11 vs 6.5 min)- decreased with practice, however experts did not show improvement after 5th attempt
Microsurgery	Malzone et al	2023	Symani Surgical System	Rat femoral vessels conventional vs robotic	End-to-end arterial and venous anastomosis	Not specified	Rat vessel diameter 1.09 mm average Procedure performance time higher in robotic group Plateau in learning curve at 60 sutures Mean number of sutures/anastomoses = 8 in both manual and robotic groups Equivalent vessel patency with histologically assessed lower tissue damage for robotic
Flap	Zhu et al	2016	Omega 6, Force Dimension, Nyon	Sheep mandible *N* = 6 Conventional vs Robot assisted	Free fibula flap—osteotomy robot assisted guidance for osteotomy line and bony fixation. Manual harvest and inset	Expert surgeon	Higher accuracy and improved implant orientation compared to freehand measurement/technique
Flap	Manrique et al	2020	Da Vinci Xi	Cadaveric *N* = 8	Bilateral DIEP pedicle dissection (TAPP and TEP approach)	Expert surgeon	Duration: TEP 56 min, TAPP 65 min Mean pedicle dissection TEP 39 min, TAPP 36 min Demonstrated feasibility, with TEP representing a less invasive technique
Abdominal wall	Sanchez et al	2018	Da Vinci	Synthetic training model Laparoscopic vs Robotic *N* = 14 (1 performed by each surgeon)	Incisional hernia repair	14 expert surgeons	Less upper limb disturbance and lower mental effort for robotic group
TOR	Chen et al	2017	Da Vinci Sp/Si	Cadaveric *N* = 4	Transoral base of tongue resection	Expert surgeon	Single port system allows for more streamlined workflow
TOR	Tay et al	2018	Endomaster	Cadaveric *N* = 4	Radical tonsillectomies	Expert surgeon	Good visualisation, quick docking
Miscellaneous	Friedrich et al	2018	Da Vinci	Silicone bench model Manual vs Laparoscopic vs Robotic *N* = 15 (for each)	Assessment of Haptic Feedback correctly order silicone with defined rigidity and 5 steel tension springs	Expert surgeon	Manual model demonstrated higher rate of correctly performed task
Education – 18 articles							
Suturing	Leijte et al	2020	RobotiX Mentor VR simulator	VR simulator	Suturing	15 Robotic surgeons 26 Laparoscopic surgeons 29 Novices	Time, economy of movement/error, accuracy, precision assessed through RobotiX Qualitative feedback reported good didactic value for proficiency-based training
Suturing	De Groote et al	2022	Not specified	Chicken Proficiency based progression training vs traditional training *N* = 18 (each arm)	Suturing/knot tying	36 novices	Higher rate of competency achieved with PBP group (eLearning until proficiency prior to task completion)
Microsurgery	Liverneaux et al	2013	Da Vinci	VR simulator, earthworm, rat model	Anastomosis	Surgical trainees	Description of training course involving 3 tier model approach with validated Structured Assessment of Robotic Microsurgery Skills (SARMS)
Microsurgery	Perez et al	2013	Da Vinci Trainer	VR exercise *N* = 49	VR exercise	11 trainees with microsurgery experience 38 trainees without microsurgery experience	Quantitative assessment: microsurgery trainees achieved better results regarding economy of movement, precision, and force Qualitative feedback: microsurgical trainees reported similar ergonomics between microsurgery and robotics
Microsurgery	Alrasheed et al	2014	Da Vinci	3 mm synthetic vessel *N* = 5 performed by each participant	End-to-end anastomosis	10 trainees	Structured Assessment of Robotic Microsurgical Skills (SARMS) assessed by 4 expert surgeons Operative Time (9-44 min) Decrease in operative time over 5 performed procedures
Microsurgery	Selber et al	2014	Da Vinci	Synthetic vessel *N* = 5 (performed by each participant in each arm) Robotic only	End-to-end anastomosis	10 surgical trainees	All skill and overall performance improved over 5 sessions, and operative time decreased for all Initially steep skill learning curve followed by gradual improvement
Microsurgery	Willems et al	2016	Da Vinci	Synthetic vessel *N* = 80 performed by each participant ( at depths of 0, 10, 20 cm with sidewall angles of 20 and 30 degrees conventional vs robotic	End-to-end anastomosis	2 surgical trainees	OSAT—no difference between manual and robotic longer duration in manual group higher subjective comfort in robotic group robotic group performed better as depth increased
Microsurgery	Clarke et al	2018		Rat vessel aorta *N* = 6 (by each surgeon in each arm) Conventional vs robotic	End-to-end anastomosis	14 microsurgeon with no robotic experience 14 robotic surgeons with no microsurgical experience	Manual Group: 17 min (microsurgeon) 44 min (robotic) Robotic Group: 37.5 min (microsurgeon) 48.5 min (robotic surgeon) Steeper learning curve with microsurgeon Feasible skill acquisition exercise
Microsurgery	Van Mulken et al	2018	Microsure	2 mm silicone vessels *N* = 10 (each arm, and by each of the participants) conventional vs robotic	End-to-end anastomosis	3 various level trainees	Anastomosis time manual vs robotic (12.5 vs 35.1 min) Comparable rate of improvement between manual and robotic when assessed with Structured Assessment of Microsurgical Skills Demonstrated steeper learning curve with the robotic group
Microsurgery	Yang et al	2022	Da Vinci Trainer	VR exercise *N* = 60	VR exercise	30 trainees with da Vinci training 30 trainees with Da Vinci training and microsurgery training	Microsurgery aided memory retention, with steeper learning curves and better skill level
Microsurgery	Beier et al	2023	Symani Surgical System	Synthetic 1/2 mm vessels / Chicken *N* = 10	End-to-end anastomosis	Expert surgeons	4-week training programme in which 10 successful anastomosis was deemed to be sufficient for progression into clinical practice
Flap	Louis et al	2017	Da Vinci Si	Porcine *N* = 3	RAM harvest	Expert surgeon	4 trocars used 80 min average operative time 16 cm average muscle length Demonstrated learning curve reflected in reduced operating time
Abdominal wall	Thomaier et al	2016	Da Vinci Trainer	Bench model *N* = 20 laparoscopic box trainer *N* = 20 robotic simulation	Peg transfer tasks	Novices	Assessment through OSATS, Global Operative Assessment of Laparoscopic Skills (GOALS) and Global Evaluative Assessment of Robotic Skills (GEARS Skill acquisition and retention following time No differences between groups after first training session. Robotic training group demonstrated higher economy of motion, and fewer errors in comparison to laparoscopic, with no significant deterioration over time
Abdominal wall	Orlando et al	2017	Da Vinci Trainer	Bench model *N* = 20 laparoscopic *N* = 20 robotic simulation	Peg transfer tasks	Novices	Assessment through OSATS, GOALS, GEARS Skill acquisition and retention following time No differences between groups after first training session. Robotic training group demonstrated higher economy of motion, and fewer errors in comparison to laparoscopic, with no significant deterioration over time
Abdominal wall	Jacob et al	2017	Da Vinci Xi	Porcine *N* = 1	Extended total extraperitoneal dissection	Expert surgeons	Successful completion of abdominal wall dissection
Mastectomy	Lee et al	2021	Da Vinci Si/Xi	Cadaveric/animal *N* = 24	Nipple-sparing mastectomy	2 Plastic surgeons 13 breast surgeons	Subjective participant feedback indicated positive learning experience
TOR	Bur et al	2017	Da Vinci	Synthetic Porcine *N* = 29	Posterior hemi glossectomy	20 surgical trainees 5 expert Surgeons	GEARS Faster performance and better technical skill in more senior surgeons Increase in scores and speed of operating over time Good qualitative feedback from trainees as a teaching model
TOR	Zhang et al	2017	Da Vinci Trainer	VR Simulator *N* = 16	12 simulated exercises	Novices	Article validates simulation training in robotic skills, with all novices achieving competency (benchmark 91%) A longer gap between training resulted in a longer time to achieve competency

**Fig. 3:**
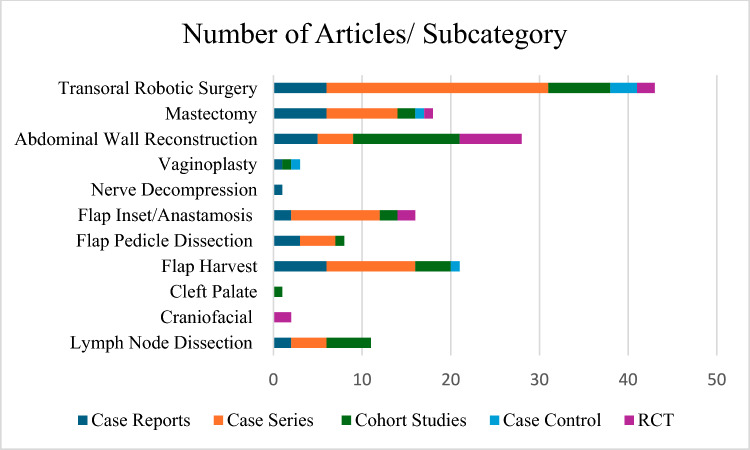
Total number of articles in each subcategory within the scope of robotic plastic and reconstructive surgery

Clinical articles were subcategorised by subspeciality (Fig. 3). A total of 11 articles described robotic lymph node dissection [13, 42–51]. A total of 21 articles described robotic pedicled or free flap harvest [52–72]. A total of eight articles described robotic flap pedicle or vessel dissection [73–80]. 16 articles detailed robotic free flap inset or anastomosis (vessel, nerve and lymphovascular) [34, 81–95]. Two articles described robotic craniofacial techniques (mandibular contouring) [96, 97]. One cohort study described a robotic cleft palate surgery [98]. One case report described robotic nerve decompression [99]. Three articles described vaginoplasty/gender reassignment robotic techniques [100–102]. A total of 28 articles described ventral abdominal wall reconstruction and hernia repair [103–130]. A total of 18 articles pertained to robotic mastectomy [56, 72, 131–146]. Finally, a total of 43 articles described transoral robotic surgery (TOR) [81, 147–188].

### Peri-operative outcomes

#### Lymph node dissection

Reported length of stay, complications, and recurrence (of disease) are displayed in Table 3. Six articles found the average operative time to be higher for robotic surgery (Table 1). The peri-operative complication rate was found to be comparable, within the reported studies. The average length of stay was shorter for robotic surgery; however, only two articles reported length of stay for conventional lymph node dissection (*P* = 0.46).

**Table 3:** Lymph node dissection length of stay, complications, and rate of recurrence within the literature

Robotic vs conventional	Control procedure	Number		Length of stay (days)		Complications		Complication rate		Recurrence		Recurrence rate	
		Robot	Control	Robot	Control	Robot	Control	Robot	Control	Robot	Control	Robot	Control
Kim et al., [42]	Endoscopic neck dissection	2	3										
Tae et al., [43]	Conventional	11	19			3	7	27.27%	36.9%	0	2	0.00%	10.53%
Kim et al., [42]	Conventional	20	33	17	15.5	6	9	30.00%	27.27%	0	2	0.00%	6.06%
Singh et al., [49]	Conventional inguinal dissection	51	100							0	0	0.00%	
Paek et al., [50]	Conventional neck dissection	28	117	4.5	4.1								
		Total	Total	Average		Total		Average complication rate		Total		Overall rate	Overall rate
		112	272	10.75	10.75	9	16	28.64%	32.10%	0	4	0.00%	7.69%
Un- paired single tail *T* test (5%)				*P* = 0.46		*P* = 0.096		*P* = 0.281		*P* = 0.00*		P = 0.008*	

Robotic only	Number	Length of stay (Days)	Complications	Complication rate	Recurrence	Recurrence rate
Lira et al., [46]	6	5			4	66.67%
Lee et al., [13]	3	8	0			
Du et al., [45]	1	6	0		0	0.00%
He et al., [48]	13		0		0	0.00%
Melly et al., [47]	1	3				
Song et al., [51]	4		1	25.00%	0	0.00%
	Total	Average	Total	Average complication rate	Total	Overall rate
	28	5.50	1	25.00%	4	16.67%

Overall											
Number of patients		Average length of stay (Days)		Complications		Average complication rate		Recurrence (total N)		Recurrence rate %	
Robot	Control	Robot	Control	Robot	Control	Robot	Control	Robot	Control	Robot	Control
**140**	272	7.25	9.80	10	16	27.42%	31.10%	4	4	3.77%	7.69%

### Pedicled and free flap harvest

Peri-operative outcomes regarding pedicled and free flap harvest are reported in Table 4. Average harvest time is higher in the robotic group, although not this was not statistically significant. Average length of stay within comparative studies is lower in the robotic group; however, overall results show a comparable length of stay with conventional surgery. Overall, average complication rates are lower than conventional approaches; however, not statistically significant within comparative studies (*P* = 0.061).

**Table 4:** Peri-operative outcomes of robotic and robotic-assisted pedicled and free flap harvest (RFFF radial forearm free flap, *RAM* rectus abdominis muscle, *LD* latissimus dorsi)

Robotic vs conventional	Control procedure	Number of patients		Length of stay (days)		Complications		Complication rate		Harvest time (min)	
		Robot	Control	Robot	Control	Robot	Control	Robot	Control	Robot	Control
Shin et al., [71]. (RFFF)	Conventional	11	11			0	1	0.00%	9.09%	107.2	67
Davila et al., [68] (RAM)	Conventional	16	20	10.2	11.2	5	11	31.25%	55.00%		
Winocour et al., [63] (LD)	Conventional	25	27	2	3	4	1	16.00%	3.00%		
Houvenaegel et al., [58] (LD)	Conventional	46	59	4.3	3.86	14	34	30.43%	57.60%		
Clemens et al., [53] (LD)	Conventional	12	64	2.7	3.4	2	24	16.67%	37.50%	92	58
Eo et al., [70] (LD)	Endoscopic/Conventional	20	37	10.2	10.8	5	9	25.00%	24.32%	75.9	35.5
		Total		Average		Total		Average Complication Rate		Average Harvest Time	
		130	218	5.88	6.45	30	80	19.89%	36.70%	91.7	53.50
Un- paired single tail *T* test				*P* = 0.415		*P* = 0.088		*P* = 0.061		*P* = 0.057	

Robotic Only	Number of patients	Length of stay	Complications	Complication rate	Harvest time
	Robot	Robot	Robot	Robot	Robot
Ozkan et al., [57] (Omental)	1	12	0	0.00%	60
Pederson et al., 2014 (RAM)	10		1	10.00%	60
Day et al., [65] (Omental)	1		0	0.00%	
Lai et al., [72] (LD)	1		0	0.00%	
Moon et al., [62] (LD)	21	7	4	19.05%	58
Cheon et al., [67] (LD)	41	9	17	41.46%	70
Chung et al., [155] (LD)	12		0	0.00%	85.8
Hwang et al., [69] (LD)	3		0	0.00%	59
Fouarge et al., [59] (LD)	6	5	0	0.00%	110
Joo et al., [66] (LD)	1	6	0	0.00%	100
Haverland et al., [61] (RAM)	6		1	16.67%	
Asaad et al., [64] (RAM)	7	7	1	14.29%	
Frey et al., [60] (Omental)	5	5.2	2	40.00%	
	Total	Average	Total	Average complication rate	Average harvest time
	115	7.31	26	10.88%	75.35

Overall									
Number of patients		Average length of stay		Total complications		Average complication rate		Average harvest time	
Robot	Control	Robot	Control	Robot	Control	Robot	Control	Robot	Control
**245**	218	6.72	6.45	56	80	13.73%	36.70%	79.81	53.50

### Microsurgery

Peri-operative outcomes for flap pedicle dissection, flap inset, and microsurgical anastomosis are shown in Table 5. No comparative studies were found for pedicle dissection, with majority of articles pertaining to deep inferior epigastric perforator (DIEP) pedicle dissection. Anastomosis time was found to be longer for robotic surgery; however, docking time was not reported in any studies. There was a comparable rate of overall complications. Only three non-comparative studies reviewed length of stay, with the average being 7.1 days.

**Table 5:** Peri-operative outcomes of robotic pedicle dissection and microsurgery. (*DIEP* deep inferior epigastric perforator)

Flap Pedicle Dissection								
Robotic Only	Number of patients	Length of stay (days)	Complications	Complication rate	Procedure time (mins)	Docking time (mins)	Harvest time (mins)	Console time (mins)
Zanaty et al., [80] (Thoracic artery)	1							
Gundlapalli et al., [73] (DIEP)	1		0	0.0%	480	20	40	
Dayaratna et al., [77] (DIEP)	1		0	0.0%	680	16	92	92
Bishop et al., [75] (DIEP)	21	3.8	5	23.8%	425.3		44.8	44.8
Daar et al., [75] (DIEP)	4	3.7	2	50.0%	717.6			
Wittesaele et al., [78] (DIEP)	10	4.5	1	10.0%	479	27.5	86	86
Tsai et al., [79] (DIEP)	13		1	7.7%		15	53	53
Choi et al., [74] (DIEP)	17				487		65	65
	Total	Average	Total	Average complication rate	Average	Average	Average	Average
	68	4	9	15.3%	545	20	63	68

Flap inset/microsurgical anastomosis								
Robotic Only	Number of patients	Length of stay	Complications	Complication rate	Procedure time (mins)	Inset time (mins)	Anastomosis time (mins)	Console time (mins)
Hans et al., [81] (RFFF inset)	1	14	0	0%	310	35		75
Song et al., [82] (RFF inset/anastomosis)	5		0	0%	591		150	
Lai et al., [83] (RFF inset, venous anastomosis)	5		0	0.0%	142	31	40	
Miyamato et al., [84] (Nerve)	6		0	0.0%				
Chang et al., [88] (Nerve graft)	1	4	0	0.0%				
Lindenblatt et al., [90] (LVA/arterial anastomosis)	5							
Beier et al., [34] (arterial anastomosis)	23		6	18.75%			69	69
Besmens et al., [92] (arterial anastomosis)	6						33	33
Chen et al., [93] (nerve anastomosis)	23	3.2	2	13.0%	510			
Innocenti et al., [94] (arterial/venous anastomosis)	1		0	0.0%			22	22
Weinzierl et al., [95] (LVA)	8		0	0%			22.6	22.6
	Total	Average	Total	Average complication rate	Average	Average	Average	Average
	76	7.07	8	3.5%	388	33	63	50

Flap inset/microsurgical anastomosis																
Robotic Vs Conventional	Control	Number of patients		Length of stay		Complications		Complication rate		Procedure time (mins)		Inset time (mins)	Anastomosis time (mins)		Console time (mins)	
		Robot	Control	Robot	Control	Robot	Control	Robot	Control	Robot	Control		Robot	Control	Robot	Control
Tsai et al., [85] (RFF inset, secondary venous anastomosis)	Conventional	14	33			2	1	15.4%	3.03%							
Lai et al., [86] (arterial/venous anastomosis)	Conventional	15	26			1	0	6.67%					38	28	38	28
Van Mulken et al., [87] (LVA)	Conventional	20	12			0	0	0%		115	81		25	9		
Barbon et al., [89] (arterial/venous/nerve/lymph anastomosis)	Conventional	22	11												25.3	14.1
Van mulken et al., [91] (LVA)	Conventional	20	12													
		Total		Total		Total		Average Complication Rate		Average			Average		Average	
		91	94	0		3	1	3.3%	3.03%	115	81.0		32	18.5	32	21.1

Overall (anastomosis/inset)															
Total number of patients		Average length of stay (days)		Total complications		Average complication rate		Average procedure time		Average inset time		Average anastomosis time		Average console time	
Robot	Control	Robot	Control	Robot	Control	Robot	Control	Robot	Control	Robot	Control	Robot	Control	Robot	Control
167	94	7.07		11	1	3.40%	3.03%	409.01	81.00	33.00		49.95	18.50	37.56	28.00

### Mastectomy

**Fig. 4:**
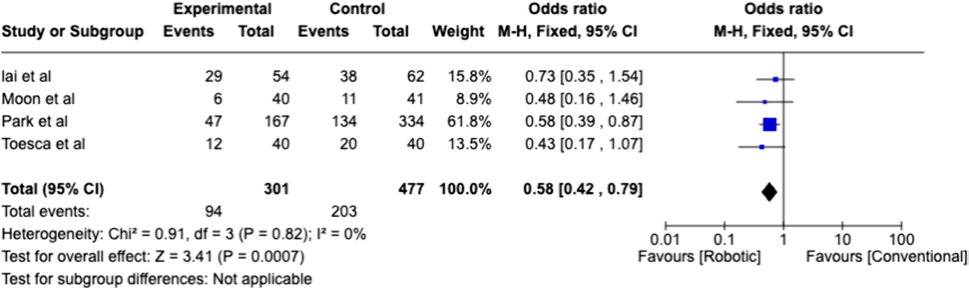
Weighted analysis of comparative studies reviewing complication rate of robotic nipple sparing mastectomy with conventional nipple sparing mastectomy

Peri-operative outcomes regarding nipple-sparing mastectomy are shown in Table 6. Operative time was found to be comparable overall; however, this included reconstruction time. Overall length of stay was comparable between open and robotic groups. Overall rate of complication was lower in robotic nipple-sparing mastectomy (*P* = 0.0007) (Fig. 4).

**Table 6:** Peri-operative outcomes in robotic nipple-sparing mastectomy

Robotic vs Conventional	Control procedure	Number		Length of stay (days)		Complications		Complication rate		Procedure time (resection alone)		Procedure time + implant		Recurrence		Recurrence rate	
		Robot	Control	Robot	Control	Robot	Control	Robot	Control	Robot	Control	Robot	Control	Robot	Control	Robot	Control
Toesca et al., [144]	Conventional	40	40	2.3	2.4	12	20	30.0%	50%	108	60	138	216				
Moon et al., [146]	Conventional	40	41	9.2	7.1	6	11	15.0%	26.83%			279	207				
Lai et al., [143]	Conventional	54	62	7	5	29	38	53.7%	61.29%			224	197	0	5	0.00%	8.06%
Park et al., [145]	Conventional	167	334			47	132	28.1%	39.5					0	2	0.00%	0.60%
		Total		Average		Total		Average complication rate		Average		Average		Total		Overall rate	
		301	477	6.17	4.83	94	208	31.7%	44.41%	108	60.0	214	206.7	0	7.0	0.0%	1.8%
Un-paired single tale *T* test				*P* = 0.307		*P* = 0.198		*P* = 0.144				*P* = 0.437		*P* = 0.072		*P* = 0.183	

Robotics only		Number	Length of stay (days)	Complications	Complication rate	Procedure time	Procedure time + implant	Recurrence	Recurrence rate
		Robot	Robot	Robot	Robot	Robot	Robot	Robot	Robot
Kuo et al., [140]	-	3	10			94		0	0.00%
Sarfati et al., [131]		1	5	0	0.0%		150	0	0.00%
Lai et al., [134]	-	2		1	50.0%			0	0.00%
Toesca et al., [132]		3	2	0	0.0%		285		
Rajappa et al., [136]	-	1	2	0	0.0%		330		
Houvenaeghal et al., [56]		44	3	26	59.1%	154			
Houvenaeghal et al., [139]	-	80	4	46	57.5%	305			
Park et al., [135]		1		0	0.0%	409		0	0.00%
Lai et al., [142]	-	39	6.7	12	30.8%		257		
Sarfati et al., [137]		33		0	0.0%		85		
Toesca et al., [132]	-	29	2	0	0.0%		180		
Lai et al., [141]		22		0	0.0%		192		
Lai et al., [72]	-	15	6.7	3	20.0%		282		
Sarfati et al., [137]		1							
		Total	Total	Total	Average Complication Rate	Average	Average	Total	Overall Rate
		274	41.4	88	18.1%	241	220	0	0.0%

Overall															
Number		Average length of stay		Total complications		Average complication rate		Average procedure time		Average procedure time + implant		Total recurrence		Recurrence rate	
Robot	Control	Robot	Control	Robot	Control	Robot	Control	Robot	Control	Robot	Control	Robot	Control	Robot	Control
**575**	477	4.99	4.83	182	208	21.5%	44.41%	214	60	214	206.7		7	0.0%	1.8%

### Abdominal wall

**Fig. 5:**
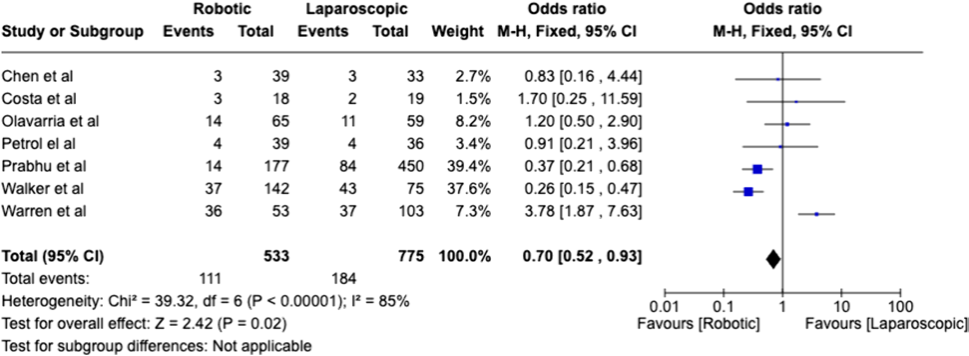
Weighted analysis of comparative studies reviewing complication rate between robotic abdominal wall reconstruction and laparoscopic abdominal wall reconstruction

**Fig. 6:**
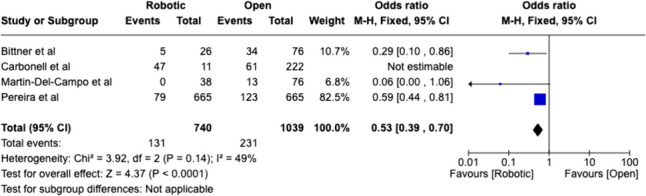
Weighted analysis of comparative studies reviewing complication rates in robotic versus open abdominal wall reconstruction

Outcomes regarding abdominal wall reconstruction are collated in Table 7. Separate comparisons are demonstrated between robotic versus laparoscopic, and robotic versus open repair. Weighted analysis of comparative robotic versus laparoscopic studies found high heterogeneity (85%), and favours robotic surgery with reduced complications (*P* = 0.02) (Fig. 5). Robotic surgery had fewer complications when compared with open surgery (*P* = 0.0001), with lower heterogeneity (Fig. 6).

**Table 7:** Peri-operative outcomes reported in abdominal wall reconstruction. (*TAR* transversus abdominis release)

Robotic vs Laparoscopic Hernia Repair	Control Procedure	Number of Patients		Length of Stay (Days)		Complications		Complication Rate		Operative Time (mins)	
		Robot	Control	Robot	Control	Robot	Control	Robot	Control	Robot	Control
Costa et al., [122]	Laparoscopic	18	19	3.67	3.95	3	2	16.67%	10.53%	355.6	293.5
Chen et al., [103]	Laparoscopic	39	33	0.49	0.21	3	3	7.69%	9.09%	156	65
Petro et al., [118]	Laparoscopic	39	36	0.5	1	4	4	10.26%	11.11%	146	94
Olavarria et al., [116]	Laparoscopic	65	59	0	0	14	11	21.54%	18.64%	141	77
Walker et al., [113]	Laparoscopic	142	75	1.4	0.7	37	43	26.06%	57.33%	116.9	98.7
Warren et al., [109]	Laparoscopic	53	103	1	2	36	37	67.92%	35.92%	245	122
Kakela et a, [123]	Laparoscopic	19	19	0.9	0.6			0.00%	0.00%	135	43.6
Prabhu et al., [107]	Laparoscopic	177	450	0	1	14	84	7.91%	18.67%	*N* = 47 < 2 h)	*N* = 31 > 2 h
		Total		Average		Total		Average Complication Rate		Average	
		552	794	0.995	1.18	111	184	19.76%	20.16%	185	113
Un-paired single tail *T* test				*P* = 0.382		*P* = 0.216		*P* = 0.484		*P* = 0.069	

Robotic vs Open	Control procedure	Number		Length of stay (days)		Complications		Complication rate		Operative time (mins)	
		Robot	Control	Robot	Control	Robot	Control	Robot	Control	Robot	Control
Martin-Del-Campo et al., [111] (TAR)	Open	38	76	1.3	6	0	13	0.00%	17.11%	299	211
Pereira et al., [126]	Open	665	665	1	3	79	123	11.88%	18.50%		
Bittner et al., [104] (TAR)	Open	26	76	3	6	5	34	19.23%	44.74%	365	287
Carbonell et al., [110]	Open	111	222	2	3	47	61	42.34%	27.48%	45% > 240	
		Total		Average		Total		Average complication rate		Average	
		840	1039	1.83	4.50	131	231	18.36%	26.95%	332	249
Un-paired single tail *T* test				*P* = 0.017*		*P* = 0.220		*P* = 0.231		*P* = 0.120	

Robotics only	Number of patients	Length of stay (days)	Complications	Complication rate	Operative time (min)
	Robot	Robot	Robot	Robot	Robot
Wang et al., [108]	1	4	0	0.00%	
Jamshidian et al., [106]	3	1	0	0.00%	88
Kudsi et al., [114] (TAR)	1	1	0	0.00%	302
Muysoms et al., [112]	41				114
Shimada et al., [127]	1	5	0	0.00%	253
Lima et al., [129]	1		0	0.00%	
Bergholz et al., [119]	1	1	0	0.00%	
Gonzalez et al., [105]	368	1	44	11.96%	102.1
	Total	Average	Total	Average Complication Rate	Average
	417	2.17	44	1.71%	172

Overall									
Total number of patients		Average length of stay (days)		Total complications		Average complication rate		Average operative time (mins)	
Robot	Control	Robot	Control	Robot	Control	Robot	Control	Robot	Control
1809	1833	1.57	2.29	286	415	12.81%	22.43%	201	144

Length of stay was shorter for robotic surgery in comparison to both groups, however, was only statistically significant for robotic versus open (*P* = 0.017). Overall operative time was higher for robotic surgery but was not statistically significant within laparoscopic and open subgroups.

### Transoral robotic surgery

TOR operative outcomes are reported in Table 8. Length of stay was shorter for robotic surgery; however, this was not statistically significant. A statistically significant lower rate of complications is found for robotic surgery in comparison to open surgery (*P* = 0.033). Disease-free survival was higher within the robotic cohort; however, this was not found to be statistically significant.

**Table 8:** Peri-operative outcomes reported for transoral robotic surgery (TOR) (DFS; disease-free survival)

Robotic Vs Conventional	Control procedure	Number of patients		Length of stay (Days)		Complications		Complication rate		DFS	
		Robot	Control	Robot	Control	Robot	Control	Robot	Control	Robot	Control
Li et al., [178]	Open	2224	6697	4.3	5.1						
Chung et al., [54] (anterior partial glossectomy)	Open	68	3915	4.8	4	0	131	0.0%	3.3%		
Chung et al., [54] (posterior pharyngectomy)	Open	641	1426	3.7	5.2	21	71	3.3%	5.0%		
Chung et al., [54] (posterior partial glossectomy)	Open	147	747	3.54	5.06	14	37	9.5%	5.0%		
Sano et al., [185]	Open	68	236								
Ford et al., [157]	Open	65	65							89%	73%
White et al., [154]	Open	64	64	3.8	8	26	55	40.6%	85.9%	74%	43%
Lee et al., [151]	Open	27	30	14.6	24.6					95.70%	91.60%
Hammoudi et al., [158]	Open	26	26	11	19	1	2				
		Total		Average		Total		Average complication rate		Average rate of DFS	
		3330	13,206	6.53	10.14	62	296	13.36%	24.80%	86.2%	69.2%
Un-paired, single tail *T* test (5%)				0.164		0.033*		0.314		0.126	

Robotic Vs Radiotherapy	Control procedure	Number of patients		Length of stay (days)		Complications		Complication rate		DFS	
		Robot	Control	Robot	Control	Robot	Control	Robot	Control	Robot	Control
Mahmoud et al., [173]	Radiotherapy	559	1314								
Nichols et al., [181]	Radiotherapy	34	34			94	74	276.5%	217.6%		
Smith et al., [165]	CRT	42	38							94%	85%
Nichols et al., [187]	Radiotherapy	34	34							88.20%	
		Total				Total		Average Complication Rate		Average DFS Rate	
		669	1420			94	74	276.47%	217.65%	91.1%	85%

Robotic Only	Number	Length of stay (days)	Complications	Complication rate	DFS	Recurrence	Recurrence rate
Van Loon et al., [159]	18	4.2	2	11.1%	86%	2	11.1%
Mercante et al., [162]	13	7	4	30.8%			
Sethia et al., [175]	111					9	8.1%
Chan et al., [147]	4	4.25	0	0.0%			
Chia et al., [148]	2015		205	10.2%			
Durmus et al., [149]	22		0	0.0%			
Durmus et al., [150]	3						
Hans et al., [81]	3	14	0	0.0%		0	0.0%
Patel et al., [152]	47		5	10.6%			
Tsang et al., 2013	1		0	0.0%			
Almeida et al., [160]	410				94.50%	43	10.5%
Dabas et al., [161]	60	4.15	3	5.0%	64%		
Razafindranaly et al., [164]	84		37	44.0%		2	2.4%
Aubry et al., [166]	178	12.6	87	48.9%			
Fujiwara et al., [167]	10	8	0	0.0%		1	10.0%
Graneli et al., [168]	1	3	0	0.0%		0	0.0%
Duek et al., [169]	1	3	0	0.0%		0	0.0%
Lallemant et al., [172]	23	12.7					
Rubek et al., [174]	30	5.3	6	20.0%			
Doazan et al., [177]	122				90.20%	14	11.5%
Scott-Wittenbom et al., [179]	6						
Hardy et al., [180]	1		0	0.0%		0	0.0%
Petruzzi et al., [182]	1		0	0.0%			
Holcomb et al., [183]	2		0	0.0%		1	50.0%
Kubik et al., [184]	23		1	4.3%			
D’Andrea et al., [186]	53		21	39.6%	46.10%		
Virgilio et al., [188]	139				71.30%		
Durmus et al. [156]	22		0	0.0%			
Frenkel et al., [170]	425		273	64.2%			
Gorphe et al., [171]	27		8	29.6%			
Mockelmann et al., [163]	41	8	9	22.0%			
Allessandrini et al., [176]	16	6.13		0.0%			
	Total	Average	Total	Average complication rate	Average DFS	Average recurrence	Overall recurrence rate
	3912	7.10	661	13.6%	75.35%	6.5	9.44%

Overall													
Number of patients		Length of stay		Complications		Average complication rate		Average DFS rate		Average recurrence		Overall recurrence rate	
Robot	Control	Robot	Control	Robot	Control	Robot	Control	Robot	Control	Robot	Control	Robot	Control
7911	14,626	6.90	10.14	817	370	22.3%	63.37%	81.18%	73.15%	6.5		9.44%	

Operative time was variable, and few conclusions can be drawn (Table 1). Lee et al. reported a longer duration compared to transoral resection; however, White et al., found a shorter duration for excision of recurrent oropharyngeal SCC [151, 154]. White et al., also found a better rate of negative margins with robotic surgery [154]. Hammoudi et al. found no difference in procedure duration for resection of primary SCC [158].

### Post-operative outcomes

Patient-reported outcomes and long-term outcomes are reported in Table 1. The quality and standard of assessment varied greatly. Patient satisfaction was reported in three (27%) lymph node (neck) dissection articles, all of which found better scores compared to open with regard to cosmesis and scarring [42–44]. Lin et al. found comparable results for patient satisfaction and pain for mandibular contouring [96].

### Flap/microsurgery

High patient satisfaction for latissimus dorsi muscle flap harvests were reported in three articles; one cohort study found significantly higher BREAST-Q scores than open [54, 66, 70]. 31% of flap inset or anastomosis articles reported post-operative outcomes other than complications [84, 85, 87, 91, 93]. Van Mulken et al. reported robotic lymphovascular anastomosis to have comparable lymph ICF scores to conventional microsurgery. Miyamoto et al. and Chen et al. reported successful patient outcomes of nerve grafts (sympathetic trunk reconstruction and nerve to deltoid) [84, 93]. Two articles detailing pedicle dissection of DIEP flaps reported favourable outcomes, and no hernias; however, there are no comparative results [73, 77].

### Abdominal wall reconstruction

Patient-reported outcome measures (PROMs) were described in 8 (28.6%) articles of abdominal wall reconstruction. Three articles reviewed pain with VAS scores and found no difference (2 RCT’s) or less pain at 1 month/1 year (prospective cohort) [116, 120, 123]. Kakela et al. found comparable PROMs (SF-36) with laparoscopic surgery, with high scores for emotional status and social function for robotic surgery. Three articles found no difference between robotic and laparoscopic surgery in reported patient outcomes, including functional status [120, 122, 126]. One RCT found higher HERqLess scores for robotic versus laparoscopic ventral mesh hernia repair [118].

One RCT compared robotic extraperitoneal versus intraperitoneal onlay mesh (IPOM) for ventral hernia repair and found that IPOM had significantly higher HerQLess scores at 1 year follow-up.

### Mastectomy

A total of four (22.2%) of mastectomy articles reported patient qualitative outcomes. Two articles reported high scores for cosmetic satisfaction with minimal scarring, whilst one case control study found significantly higher scores in a cosmetic outcome questionnaire than open surgery, with better scarring and a better position of the nipple–areolar complex [132–134, 143]. One RCT documented significantly higher satisfaction within the BREAST-Q questionnaire for robotic surgery [144].

### TOR

Three TOR studies reported a lower rate of tracheostomies in the peri-operative period, as well as a lower requirement and durations of nasogastric feeding/PEG feeding [154, 155, 158].

Two studies found significantly higher 3-year disease-free survival with robotic surgery in HPV negative patients, and comparable rates of survival for HPV positive patients for oropharyngeal SCC primary resection [157, 173]. This was echoed by Lee et al., in which robotic surgery had a higher overall and disease-free survival rate at 2 years for lateral oropharyngectomy as treatment for tonsillar cancer [151]. White et al. found a higher rate of 2-year disease-free survival for open surgery to treat recurrent oropharyngeal SCC (T1-T4) [155].

Two articles evaluated patient outcomes through the Head and Neck Cancer Inventory (HCNI); Durmus et al. reported patients to have highly functional quality of life within their case series of carcinoma of unknown primary resection [156]. Sethia et al. found comparable outcomes for robotic oropharyngeal resection with and without adjuvant therapy [175]. Lee et al. also reported no difference in VHI and MDADI scores between open and robotic lateral oropharyngectomy for tonsillar cancer [151].

### Cost

Gundlapalli et al. reported a higher procedural cost for their case report of a robotic-assisted DIEP breast reconstruction of $16,000 versus $14,000. There were no other articles which reported cost within robotic flap harvest or microsurgery.

Lai et al. reported a higher cost for robotic nipple-sparing mastectomy in comparison to conventional treatment of $10, 877 versus $5,702 [143].

Within the subcategory of abdominal wall reconstruction three articles (11%) reported cost. Olavarria et al. found robotic patients had an increased total cost for 90 days of care in comparison to laparoscopic ventral mesh hernia repair in their RCT ($15, 865 robotic versus $12, 955) [116]. In addition to this, a separate RCT found that whilst the cost of reusables was comparable between robotic and laparoscopic ventral hernia repair, the total cost was significantly higher for robotic patients due to the overall operative time (Cost ratio of 1.13 robotic versus laparoscopic 0.97 *P* = 0.03) [125]. In contrast a retrospective cohort study found whilst the procedure costs were higher for robotic surgery, the overall cost of patient care was shorter because of reduced length of hospital stay (robotic $13, 943 versus $19, 532, *P* = 0.07) [109].

Two TOR articles reported cost (4.7%). Chung et al. found that overall cost was significantly lower for robotic pharyngectomy ($20,706 versus $29,365) and posterior partial glossectomy ($19, 091 versus $23,414), whilst anterior partial glossectomy demonstrated no difference in the total cost of procedure between TOR and conventional approaches ($22,111 versus $21,376) [155]. Hammoudi et al. reported higher costs for robotic oropharyngeal SCC resection; however, the overall cost accounting for duration of hospital stay was significantly less ($20,885 vs $27,926) [158].

### Learning curve

Learning curve was reported in clinical studies as changes in operative time (Table 1). Three abdominal wall reconstruction articles commented that skin-to-skin operating time decreased throughout their cohort [112, 116, 120]. Muysoms et al. analysed operative time for 41 transabdominal retromuscular hernia repairs, and commented that the decrease was largely contributed to by improved efficacy in the dissection aspect of the procedure [112]. Olavarria et al. reported a training exposure of 50 cases, through simulation and cadaveric models, prior to performing ventral hernia repairs was necessary to ensure optimal clinical practice [116]. A total of four mastectomy articles reported operative time to decrease with as clinical exposure increased, including a decrease in docking time [132–134, 139, 142]. Lai et al. achieved an average time for nipple-sparing mastectomy of 100 min, in a series of 39 patients [142].

Van Mulken et al. reported robotic microvascular anastomosis to require a longer time to complete; however, a steep learning curve resulted in a reduction in this [87]. Barbon et al. also reported a steep learning curve for anastomosis with time taken to complete being comparable to hand-sewn operative time, with the quickest robotic anastomosis taking around 10 min (Table 2) [89].

Selber et al. also reported a steep learning curve in surgical trainees over five sessions, followed by gradual improvement [29]. Two training models in microvascular anastomosis reported a plateau in learning curve of robotic anastomosis by expert surgeons on synthetic silicone vessels and rat vessels to be 5 and 8 attempts, respectively [16, 17]. Beier et al. developed a 4-week training programme with synthetic 1 and 2 mm vessels, in which 10 successful anastomosis were deemed to be the benchmark for skill acquisition before progression to clinical practice [34].

### Surgical ease of use

Robotic surgery offers several mechanical advantages to aid surgical performance. Many authors commented upon improved visibility with higher 3-dimensional resolution, magnification, and lighting, allowing for depth of field perception and a 360° view of a cavity [54, 132].

The Da Vinci robotic arms have 7° of freedom which allow for higher dexterity and greater range of motion, optimising the user’s ability to dissect the surgical plane and increasing access to difficult anatomical areas [137].

Insufflation was found to be useful attribute for nipple-sparing mastectomy [131, 135, 141]. Through a single small incision approach, Toesca et al. reported easy identification of structures such as intercostal perforators which contribute to nipple–areolar complex survival and flap survival, and better view of the surgical plane [132]. The use of carbon dioxide helped to reduce bleeding and perform better haemostasis [132]. There was a higher surgical challenge with larger ptotic breasts [136]. Motion scaling, and tremor filtration provides high precision and stability; this was also found to be advantageous for flap and microsurgery [77, 132, 137].

The robotic technique of pedicle dissection of the DIEP flap minimizes incision of the anterior rectus muscles and provides improved dexterity and motion; however, due to the space occupation of the robot and the console it may be challenging for two surgical teams to work simultaneously, thus potentially increasing operative duration [73, 77, 80].

Robotic equipment also eliminates haptic feedback; however, users have reported that they were able to compensate effectively for this by relying on visual cues and felt able to complete the vessel and lymphovascular anastomosis without difficulty [83, 85, 90].

Feng et al. reviewed tremor during microsurgery, based on instrument tip movement and found that this was significantly lower in robotic surgery in an ex vivo model [14]. Furthermore, in a simulation model of 1 mm synthetic vessels, robotic anastomosis was performed with greater precision (measured in suture distance and angulation) when compared with manual approaches for 40 expert surgeons and 20 novices [17].

## Discussion

This study demonstrates feasibility and safety of robotic surgery within plastic and reconstructive surgery in several subcategories. There are clear benefits to the surgeon, as described above, with improved access to difficult areas, tremor reduction and motion scaling, and improved ergonomic efficiency [2].

These attributes are particularly useful in cavity surgery and could create opportunities to complete challenging procedures which could not be accessed through an open approach due to narrow openings, such as nasopharyngeal resection and microvascular reconstruction, or where there may be a high risk of complications, or prolonged recovery time associated with conventional open approaches.

One example of this is TOR, whereby access and exposure is often obtained through techniques with higher morbidity, such as mandible splitting, leading to specific complications and expectations for recovery outside of the intended resection. Furthermore, although DIEP flap harvest can be regarded as having more superficial access, Tsai et al. found the anterior rectus sheath incision for pedicle dissection to be significantly smaller than conventional approaches, and thus less invasive [79]. It is not yet clear if this translates to reduced hernia occurrence post-operatively.

As interest within microsurgery grows, Da Vinci, and other companies such as Symani Surgical Systems and Microsure, have created an instrument portfolio that is well adapted to this field. Literature shows these tools can perform vessel, nerve and lymphovascular anastomosis with non-inferior outcomes to conventional approaches. Improved surgical ergonomics has allowed end-to-end anastomosis of 1 mm diameter vessels as reported in preclinical studies, with higher ease [16]. Whilst nerve repair can be performed robotically, there is lack of substantial evidence or comparison to conventional approaches. Whilst this approach is more minimally invasive, further research to determine the overall benefit, safety and cost would be beneficial.

Loss of haptic feedback is often considered to be disadvantage of robotic surgery. Surgeons have reported a compensation for this by relying on visual cues which has not impacted their performance. Further research could assess how easily a surgeon may adapt to the loss of true haptic feedback, as well as looking into the incorporation of haptic feedback into robotic instruments.

Single port access is highly advantageous for breast surgery including resection and reconstruction. Quicker docking can reduce operative time and the smaller incision offers a better cosmetic outcome with reduced scarring [101].

The high precision and accuracy of robotic surgery, could improve patient care, reflected in the lower rate of complications reported, reduced blood loss, reduced post-operative pain, as well as the comparable or reduced length of recovery. Whilst operative time is reported to be higher for robotics, many centres have shown a learning curve in adapting to new techniques.

### Post-operative outcomes

There is a paucity of data evaluating patient reported outcomes within the literature. Outcomes within case series/case reports were often reported anecdotally, without use of validated or quantitative assessment tools. However, several articles have reported high patient satisfaction with regard to cosmetic outcome and scarring. Robotic neck dissection approach has been performed with a smaller retro-auricular incision.

Furthermore, robotic latissimus dorsi muscle flap harvest and radial forearm flap harvest can offer reduced scarring through a more minimally invasive approach, resulting in absence of long scars, on the back and forearm, respectively. Whilst this is the case, compared to open techniques, insufflation with reduced scarring, can also be achieved with an endoscopic approach. A comparison of the benefits to the surgeon and patient between endoscopic and robotic-assisted technique would be valuable to ascertain the true benefit of robotic assistance in this procedure. Some patients may require incorporation of skin within an LD flap for example in salvage procedures, or delayed reconstruction of the irradiated breast. The quality of coverage at the recipient site may be insufficient to accommodate the optimal reconstructive outcome, with particular importance of the integrity of the lower pole. In these circumstances, robotic surgery may present few advantages for LD flap harvest, and thus patient selection is important.

Patients undergoing robotic nipple-sparing mastectomy and reconstruction have also reported a higher scar satisfaction, with the use of a single incision in the axilla, in which multiple robotic arms can be used. There is a clear benefit to procedures in which access can move towards less invasive approaches, and robotic surgery within breast reconstruction and lymph node dissection are promising avenues for future research.

The rate of hernia recurrence within abdominal wall reconstruction is challenging to ascertain given the variable and often short length of follow-up reported within the literature. The mean length of follow-up within this subcategory is 9 months (0.25–33.6 months).

There is a high variance of histopathology within the transoral robotic surgery subcategory, as well as tumour location, stage of disease, and patient demographics. Few conclusions can be drawn between the comparative studies given the variability. However, the results reported, suggest that TOR results in non-inferior patient outcomes in comparison to conventional approaches.

### Cost

Cost is poorly reported within the literature. Cost-analysis of robotic reconstructive procedures to review total cost of patient care would be beneficial in ascertaining the economic barriers that prevent the implementation of robotic within clinical practice in this speciality. Reasons suggested for higher cost include the initial purchase of robotic equipment, and prolonged operative duration utilising resources [118].

However, several articles within abdominal wall reconstruction and TOR, have reviewed the total cost of patient care, and found that the overall financial burden is significantly less than conventional approaches after accounting for length of hospital stay. This could be because of fewer complications, and reduced pain with a minimally invasive approach [109, 155, 158]. Chung et al. also reported reduced requirement of tracheostomies, nasogastric feeding, and percutaneous endoscopic gastrostomy (PEG) feeding, which could account for a decrease in overall consumables cost. Several articles have also described a learning curve throughout their studies, reflected in a shorter operative duration, which could have an impact for cost incurred. The cost of training surgeons, and theatre teams to use robotic equipment should also be accounted for.

Whilst the initial cost may be high for robotic surgery, the overall cost may be offset by the reduction in complication rate, and reduced length of stay. It is important to delineate when and where the cost of robotics, including resource utilisation, is balanced by proven improved patient outcomes in order to implement this effectively in future practice.

### Learning curve

All studies which report a learning curve in this review, do so indirectly, as a reduction in operative time [142]. Whilst a reduction in the time taken to perform the procedure can be seen as an improvement in skill acquisition, duration of surgery can be affected by various factors in clinical practice including team efficiency and education. Standardised training for skill acquisition with appropriate measures of assessment in a controlled setting will aid in understanding the number of procedures required to achieve clinical competency in each subspeciality. global evaluative assessment of robotic skills (GEARS), and structured assessment of robotic microsurgery skills (SARMS), have been used as objective quantitative assessment tools in this field.

Training should also encompass theatre staff, as set up time including robot docking, change of arms, and equipment troubleshooting can be optimised to reduce burden and improve patient care [133]. Prolonged operative duration incurs significant resource utilisation including time, cost, equipment, and staff. Barbon et al. was able to demonstrate a steep learning curve in microvascular anastomosis to achieve an anastomotic time which was comparable with conventional approaches [89].

Vierstraete et al. describe the current training pathway of abdominal wall reconstruction and ventral hernia repair and found in their experience of posterior component separation that there was a gradual reduction in operative time until the surgical team reached their ‘comfort zone’ at around 20–25 cases. Depending on the frequency with which this procedure is performed, it may take a long period of time for the surgeon to reach that level of experience [189].

### Other limitations

This report shows technical feasibility of robotic surgery; however, many articles are a relatively low level of evidence, with a high prevalence of case reports and case series. This review presents small sample sizes and as such, statistical analysis is likely to be underpowered, impeding ability to present true statistical significance. Whilst this study can suggest non-inferiority of robotic surgery, patient advantages remain to be clearly demonstrated.

There is a lack reported of long-term outcomes and formal PROMs, with variable follow-up duration. Due to large heterogeneity of the data and variance within patient selection, and outcomes reported, particularly within transoral robotic surgery, we have been unable to perform a weighted analysis for most subcategories, which would provide a more powerful comparison.

## Conclusions

This literature review demonstrates technical feasibility of robotics in plastic and reconstructive surgery. High cosmetic satisfaction is reported with minimally invasive approaches. Operative time is higher than conventional approaches, although steep learning curves are reported, and this may contribute to a higher initial cost. Overall cost may be offset with improved patient outcomes within TOR and abdominal wall reconstruction; however, further reporting of cost and cost-effectiveness is necessary. Technical advantages can potentially translate to improvements in complication rate, and a faster recovery time, with non-inferior patient outcomes reported, with thoughtful case selection. However clearer evidence to support improved outcomes within the field, particularly in comparison with laparoscopic surgery, is required to justify the financial incurrence and demand on resources. Robotic surgery could play an exciting role within plastic surgery, and future research should focus on robotic training, as well as producing higher quality comparative clinical research, which is adequately powered, to fully understand the true benefit for patient care.

## Supplementary Information

Below is the link to the electronic supplementary material.

Supplementary file1 (PNG 749 KB)

Supplementary file2 (PNG 2249 KB)

Supplementary file3 (PDF 26 KB)

Supplementary file4 (PDF 15 KB)

## Data Availability

No datasets were generated or analysed during the current study.
