# Supplementary figures and images for: The emerging role of robotics in plastic and reconstructive surgery: a systematic review and meta-analysis
Source: J Robot Surg. 2024 Jun 15;18(1):254. doi: 10.1007/s11701-024-01987-7 (PMC11180031; doi:10.1007/s11701-024-01987-7)

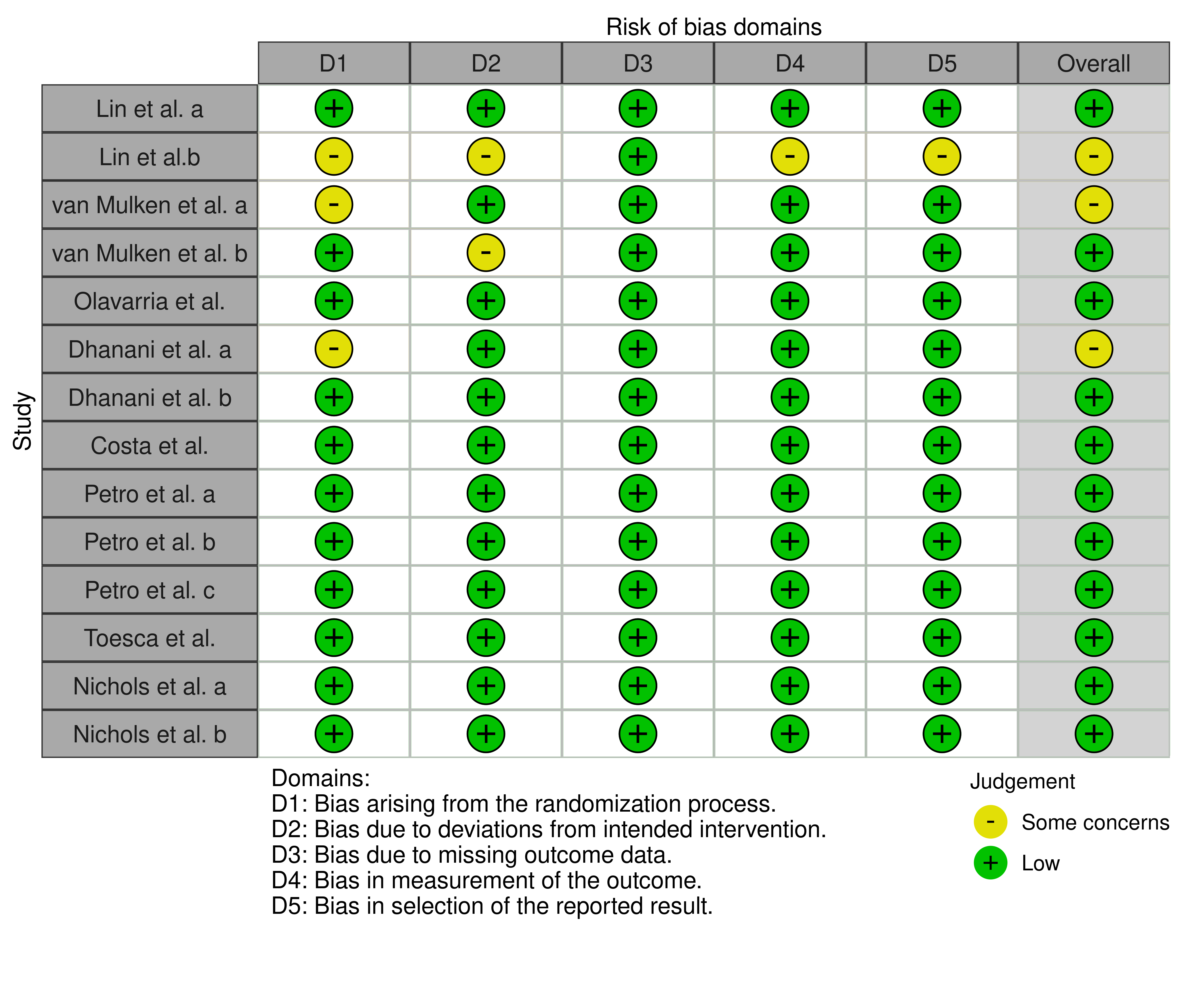

Supplement: Supplementary file 1 — Supplementary file1 (PNG 749 KB) [file 11701_2024_1987_MOESM1_ESM.png]

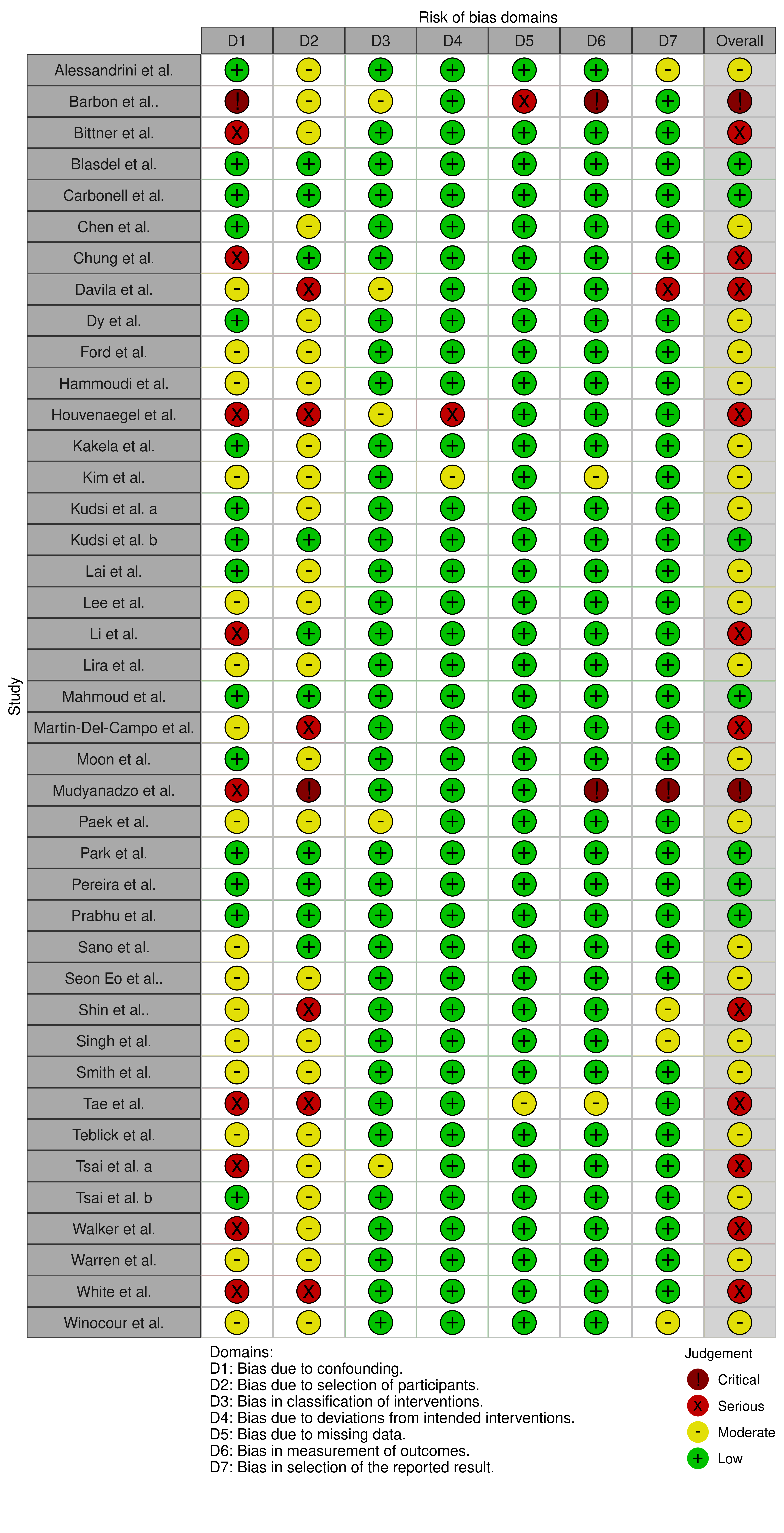

Supplement: Supplementary file 2 — Supplementary file2 (PNG 2249 KB) [file 11701_2024_1987_MOESM2_ESM.png]
