# Supplementary material for: The emerging role of robotics in plastic and reconstructive surgery: a systematic review and meta-analysis
Source: J Robot Surg. 2024 Jun 15;18(1):254. doi: 10.1007/s11701-024-01987-7 (PMC11180031; doi:10.1007/s11701-024-01987-7)
Supplement: Supplementary file 3 — Supplementary file3 (PDF 26 KB) [file 11701_2024_1987_MOESM3_ESM.pdf]

	Q1	Q2	Q3	Q4	Q5	Q6	Q7	Q8	Q9	Q10	Overall
Almeida et al.	Yes	Yes	Yes	Yes	Yes	Yes	Yes	Yes	No	Yes	Include
Asaad et al.	Yes	Yes	Unclear	Yes	Yes	Yes	Yes	Yes	No	Unclear	Include
Aubrey et al.	Yes	Yes	Yes	Yes	Yes	Yes	Yes	Yes	No	Yes	Include
Beier et al.	Yes	Yes	Yes	Unclear	Unclear	Yes	Yes	Yes	No	Yes	Include
Besmens et al.	No	Unclear	Unclear	Unclear	Unclear	Yes	Yes	Yes	No	N/A	Include
Bishop et al.	Yes	Yes	Unclear	Unclear	Unclear	No	No	No	No	Yes	Include
Chan et al.	Yes	Yes	Unclear	Unclear	Unclear	Yes	Yes	Yes	No	Yes	Include
Chen et al.	Yes	Yes	Unclear	Yes	Unclear	Yes	Yes	Yes	No	Yes	Include
Cheon et al.	Yes	Yes	Yes	Yes	Yes	Yes	Yes	Yes	No	Yes	Include
Chia et al.	Yes	No	No	No	No	No	No	Yes	No	Yes	Include
Choi et al.	Yes	Yes	Unclear	Yes	No	No	No	No	No	Yes	Include
Chung et al.	Yes	Yes	Yes	Yes	Yes	Yes	Yes	Yes	No	Yes	Include
Clemens et al.	Yes	Yes	Yes	Yes	Yes	Yes	Yes	Yes	No	Yes	Include
D'Andrea et al.	Yes	Yes	Yes	Yes	Yes	Yes	Yes	Yes	No	Yes	Include
Daar et al.	Yes	Yes	Yes	Yes	Yes	Yes	Yes	Yes	No	Yes	Include
Dabas et al.	Yes	Yes	Yes	Unclear	Yes	Yes	Yes	Yes	No	Yes	Include
Doazan et al.	Yes	Yes	Yes	Yes	Yes	Yes	Yes	Yes	No	Yes	Include
Durmus et al. a	Yes	Yes	Yes	No	Yes	Yes	Yes	Yes	No	Yes	Include
Durmus et al. b	Yes	Yes	Unclear	Unclear	Unclear	Yes	Yes	Yes	No	Yes	Include
Durmus et al. c	Yes	Yes	Yes	Yes	Yes	Yes	Yes	Yes	No	Yes	Include
Fouarge et al.	Yes	Yes	Yes	Yes	Yes	No	No	No	No	N/A	Include
Frenkel et al.	Yes	Yes	Yes	Yes	Yes	Yes	Yes	Yes	Yes	Yes	Include
Frey et al.	Yes	Yes	Unclear	Yes	Yes	Yes	No	No	No	N/A	Include
Fujiwara et al.	Yes	Yes	Yes	Unclear	No	Yes	Yes	Yes	No	Yes	Include
Gonzalez et al.	Yes	Yes	Yes	Yes	Yes	Yes	Yes	Yes	No	Yes	Include
Gorphe et al.	Yes	Yes	Yes	Yes	Yes	No	Yes	Yes	No	Yes	Include
Hans et al.	Yes	Yes	Yes	Unclear	Yes	Yes	Yes	Yes	No	N/A	Include
Haverland et al.	Yes	Yes	Yes	Yes	Yes	Yes	Yes	Yes	No	Yes	Include
He et al.	Yes	Yes	Yes	Unclear	Unclear	No	No	No	No	Yes	Include
Houvenaeghal et al. a	Yes	Yes	Yes	Yes	Yes	No	Yes	Yes	No	Yes	Include
Houvenaeghal et al. b	Yes	Yes	Unclear	Unclear	Unclear	No	Yes	Yes	No	Yes	Include
Jamshidian et al.	Yes	Yes	Yes	Yes	Yes	Yes	Yes	Yes	No	Yes	Include
Kim et al.	Yes	Yes	Yes	Unclear	Unclear	Yes	No	Yes	No	Yes	Include
Kubik et al.	Yes	Yes	Yes	Yes	Yes	Yes	Yes	Yes	No	Yes	Include
Kuo et al.	No	Unclear	Unclear	Unclear	Unclear	Yes	Yes	Yes	No	N/A	Include
Lai et al. a	Yes	Yes	Yes	Yes	Yes	Yes	Yes	Yes	No	Yes	Include
Lai et al. b	No	Unclear	Unclear	Unclear	Unclear	No	No	No	No	N/A	Include
Lai et al. c	Yes	Yes	Yes	Yes	Yes	No	Yes	Yes	No	Yes	Include
Lai et al. d	Yes	Yes	Yes	Yes	Yes	Yes	Yes	Yes	No	Yes	Include
Lai et al. e	Yes	Yes	Yes	Yes	Yes	Yes	Yes	Yes	No	Yes	Include
Lallemant et al.	Yes	Yes	Yes	Yes	Yes	Yes	Yes	Yes	No	Yes	Include
Lindenblatt et al.	Unclear	Unclear	Unclear	Unclear	Unclear	No	Yes	Yes	No	N/A	Include
Mercante et al.	Yes	Yes	Yes	Yes	Unclear	Yes	Yes	Yes	No	Yes	Include
Miyamato et al.	Unclear	Yes	Unclear	Unclear	Unclear	No	No	Yes	No	Yes	Include
Mockelmann et al.	Yes	Yes	Unclear	Unclear	Unclear	Yes	Yes	Yes	No	Yes	Include
Moon et al.	Yes	Yes	Yes	Yes	Yes	Yes	No	Yes	No	Yes	Include
Muysoms et al.	Yes	Yes	Yes	Yes	Yes	Yes	Yes	Yes	No	Yes	Include
Patel et al.	Yes	Yes	Yes	Yes	Yes	Yes	Yes	Yes	No	Yes	Include
Pederson et al.	No	Yes	Yes	Unclear	Unclear	No	No	No	No	Yes	Include
Rayman et al.	Yes	Yes	Yes	Yes	Yes	Yes	Yes	Yes	No	Yes	Include
Razafindranaly et al.	Yes	Yes	Yes	Yes	Yes	Yes	Yes	Yes	No	Yes	Include
Rubek et al.	Yes	Yes	Yes	Yes	Yes	Yes	Yes	Yes	No	Yes	Include
Safarti et al.	Yes	Yes	Yes	Yes	Yes	Yes	Yes	Yes	No	Yes	Include
Scott-Wittenborn et al.	Unclear	Unclear	Unclear	Unclear	Unclear	No	No	No	No	N/A	Include
Sethia et al.	Yes	Yes	Yes	Unclear	Unclear	Yes	Yes	Yes	No	Yes	Include
Song et al. a	No	Yes	Yes	Unclear	Unclear	Yes	Yes	No	No	Yes	Include
Song et al. b	Yes	Yes	Unclear	Yes	Yes	Yes	Yes	Yes	No	Yes	Include
Toesca et al.	Yes	Yes	Yes	Yes	Yes	Yes	Yes	Yes	No	Yes	Include
Van Loon et al.	Yes	Yes	Yes	Yes	Yes	Yes	Yes	Yes	No	Yes	Include
Virgilio et al.	Yes	Yes	Yes	Yes	Yes	Yes	Yes	Yes	No	Yes	Include
Weinzierl et al.	Unclear	Yes	Unclear	Unclear	Unclear	Yes	Yes	No	No	Yes	Include
Wittesaele et al.	Yes	Yes	Yes	Yes	Yes	Yes	Yes	Yes	No	Yes	Include
